# Supplementary material for: Evaluating the boundaries of marine biogeographic regions of the Southwestern Atlantic using halacarid mites (Halacaridae), meiobenthic organisms with a low dispersal potential
Source: Ecol Evol. 2019 Nov 7;9(23):13359–74. doi: 10.1002/ece3.5791 (PMC6912894; doi:10.1002/ece3.5791)
Supplement: Supplementary file 1 [file ECE3-9-13359-s001.pdf]

Appendix 1: Timetree inference of acariform mites (Acariformes)

Since paleontological data for marine mites are absent, we conducted a phylogenetic analysis using outgroup time calibration. A similar approach was used for feather mites resulting in time estimates largely compatible with independent dating of the avian host biogeographic events (Klimov *et al.*, 2017). Our outgroup time calibration analysis was based on previous works (Pepato & Klimov, 2015, Klimov et al., 2018, Dabert et al, 2016, Pepato et al., 2018), but we included a representative set of halacarid marine mite terminals, with all our target species. Species divergence time estimates obtained by this analysis were then used to inform the inference of coalescence times for Cytochrome Oxidase I for our species/population-level datasets.

Material and Methods

Sampling and Sequencing. Taxon names, taxonomic classification, sequenced loci (COI, 18S, 28S) and their GenBank accession numbers are listed in Table S1. For molecular work, we used previously described amplification and sequencing protocols and primers (Klimov et al., 2018, Pepato et al., 2018).

Molecular analyses. Mitochondrial COI alignment was unambiguous (no indels); stop-codons, which are indicative of pseudogenes, were not detected after amino acid translation. Alignment of rDNA was done in BioEdit 7.2.1 (Hall, 1999) based on secondary structure information (Kjer, 1995) and previous studies (Pepato & Klimov, 2015, Pepato et al, 2018).

Best-fitting models of nucleotide substitutions were found in Partition Finder 1.0.1 (Lanfear *et al* 2012) using corrected Akaike Information Criterion (AICc). COI alignment was partitioned by codon positions and rDNA by paired (e.g., stems) and non-paired (e.g., loops) regions. Saturation of each partition was tested in DAMBE *6* (Xia et al., 2017); for CO1 the third codon position was excluded from analyses due to saturation (Iss > Iss.c: Iss = 1.239; Iss.cSym = 0.692 (P << 0.0001); Iss.cAsym = 0.364 (P << 0.0001)).

Molecular clock dating was performed in BEAST 2.3.2 (Bouckaert et al., 2014). Each analysis was run in five replicates and comprised at least $2x10^8$ generations sampled every 10,000th generation. BEAST input file was generated in BEAUTi 2. Convergence of analyses was evaluated in Tracer 1.7 (Rambaut et al., 2018). Trees were visualized in FigTree 1.4.2 (http://tree.bio.ed.ac.uk/software/figtree/). Substitution models were set with the uncorrelated lognormal relaxed clock model (Drummond et al., 2006). The Yule speciation model showed a slightly better fit than the Birth and Death model using AICM, 289616.524 vs 289620.183 (calculated in Tracer). Because the former is less parameterized, it was preferred over the Birth and Death speciation model.

A lognormal prior distribution of ages was set to twelve paleontological calibration points associated with well-supported relationships, all treated as monophyletic clades (Table S2). Their offset corresponded to the minimal age and the mean calculated such that 95% of the distribution lies between the minimal ages and the soft maximum bounds, with a standard deviation of 1.00. Minimal ages correspond to the minimal estimated age of descent from a given node. It may be the absolute age of the fossiliferous strata or the minimum age of the Stage/Age to which the fossil was assigned according the International Chronostratigraphic Chart (http://www.stratigraphy.org/ICSchart/ChronostratChart2017-02.jpg, accessed June 06, 2017). Maximal soft bounds were based upon maximal age of the first occurrences of fossils attributed to a well-supported, more inclusive clade to which the node belongs. Preliminary, prior-only runs were performed to check the densities for all calibrated nodes and establish an exponential distribution to the root that reflects the fossil record. The exponential distribution on the root was established with the mean = 16.9 and offset = 405.0 Ma. Prior distributions obtained from the priors-only analysis are given in Fig. S1.

The divergence times are given as medians and 95% highest posterior densities (HPDs) as estimated in TreeAnnotator. In order to employ the distributions obtained in these analyses for our secondary calibration, stationary trees were also summarized as means, given that medians and means may be used to set lognormal distributions that provide better descriptions of the output than normal distributions (Morrison, 2008).

Results and Discussion

Our topology (Fig. S2A-B) is similar to previous analyses concerning Halacaridae ingroup phylogeny (Pepato et al., 2018). It differs, however, by grouping the genus *Anystis* as sister group of Halacaridae in a clade comprising all Anystina with the following topology ((*Andocaeculus*, *Erythracarus*) ((Halacaridae, *Anystis*), Parasitengona)). For the first time the traditional grouping of Anystina was recovered in molecular analyses, possibly due to improved sampling. The split between the Halacaridae+*Anystis* clade and Parasitengona was recovered 374.8, 319.3-432.9 Ma, a result that pushes back this divergence in time if compared to previous studies (321.5, 264.0-381.3 Ma Pepato et al., 2018; 320, 270-345 Ma, Dabert et al., 2016). In our analysis, the divergence between *Anystis* and Halacaridae occurred at 329.4, 265.0-393.2 Ma.

The internal topology of Halacaridae was identical to that reported in Pepato et al. (2018), except that it includes a representative of the freshwater genus *Porolohmannella* and excludes some terminals with large proportions of missing data. The first branching in the stem Halacaridae was the subfamily Lohmannellinae (*Scaptognathus*), occurring 269.1, 216.7-329.3 Ma, a result similar to that published previously (271.3, 221.7-324.2 Ma). Therefore, once the

transition between land and sea occurred at some point after the split from *Anystis*, the values for the age of transition from the land to the sea are similar to those recovered in Pepato et al. (2018).

Despite not being the focus of this study, it is noteworthy that the freshwater genus *Porolohmannella* was recovered as sister group to *Limnohalacarus*. It narrowed the age of freshwater transition in this lineage: in previous analyses, the divergence between the freshwater lineages and their marine ancestors occurred 219.2, 165.9-274.6 Ma. In the new analyses, the values are very similar (224.3, 151.6-245.6 Ma), but now the divergence between *Porlohmannella* and *Limnohalacarus* was 136.8, 63.1-204.8 Ma, pushing back in time the minimal age for this freshwater lineage crown group.

The resemblance in gnathosomal morphology of Pezidae (a rare Australian lineage, not available for sequencing) and *Lohmannella* and *Porolohmannella*, was the main reason of placing the freshwater family Pezidae in Halacaroidea (Harvey, 1990). Because *Porolohmannella* does not form a monophyletic group with the remaining Lohmannellinae (as expected based on overall similarity) and considering that Pezidae females share a conspicuous apomorphic character (females attach their eggs to their hind legs), our new analysis suggests that Peziidae is a very derivative Halacaridae, and not a separate family. It would be very interesting to definitively solve this evolutionary question in the future.

Our focal taxon, the *Rhombognathus levigatoides* species complex was recovered as sister species diverging from each other 7.2, 1.69-16.28 Ma. It was recovered in a weakly supported clade with the undescribed species occurring in the South of São Paulo State (PP= 0.69), and hence was not employed as a secondary calibration in downstream analyses. These three terminals were grouped with an unnamed species from California (27.1, 14.37-43.12 Ma), and then with *Rhombognathus areolatus* (39.3, 21.7-60.39 Ma). *Agauopsis legionium* diverged from its sister species from California at 43.3 (17.6, 76.7), both diverging from *Agauopsis bilophus* at 74.5 (40.7, 114.8) Ma.

References

Bouckaert, R., Heled, J., Kühnert, D., Vaughan, T., Wu, C-H., Xie, D., Suchard, M. A., Rambaut, A., & Drummond, A. J. (2014). BEAST 2: A Software Platform for Bayesian Evolutionary Analysis. *PLoS Computational Biology*, 10(4), e1003537. doi:10.1371/journal.pcbi.1003537.

Cockerell, T. D. A. (1917). Arthropods in Burmese amber. *Psyche*, 24, 40-44.

Dabert, M., Proctor, H. & Dabert, J. (2016). Higher-level molecular phylogeny of the water mites (Acariformes: Prostigmata: Parasitengonina: Hydrachnidiae). *Molecular Phylogenetics and Evolution*, 101, 75-90.

Drummond, A.J., Ho, S.Y.W., Phillips, M.J. & Rambaut, A. (2006) Relaxed phylogenetics and dating with confidence. PLoS Biology, 4, e88.

Dubinin, V. B. (1962). Class Acaromorpha: mites or gnathosomic chelicerate arthropods. In: Rodendorf, B. B. (Ed.) *Fundamentals of Palaeontology*. (pp. 447–473). Moscow: Academy of Sciences of the USSR [in Russian].

Dunlop, J. (2007). A large parasitengonid mite (Acari, Erythraeoidea) from the Early Cretaceous Crato Formation of Brazil. *Fossil Record*, 10, 91-98.

Hall, T.A. (1999). BioEdit: a user-friendly biological sequence alignment editor and analysis program for Windows 95/98/NT Nucleic Acids Symposium Series, 41, 95-98.

Kjer, K. (1995). Use of ribosomal-RNA secondary structure in phylogenetic studies to identify homologous positions—an example of alignment and data presentation from the frogs. *Molecular Phylogenetics and Evolution*, 4 (3), 314-330

Klimov, P. B., Mironov, S. V. & OConnor, B. M. (2017). Detecting ancient codispersals and host shifts by double dating of host and parasite phylogenies: Application in proctophyllodid feather mites associated with passerine birds. *Evolution*, 71, 2381-2397.

Klimov, P. B., OConnor, B. M., Chetverikov, P. E., Bolton, S. J., Pepato, A. R., Mortazavi, A. L., Tolstikov, A. V., Bauchan, G. R., Ochoa, R. (2018). Comprehensive phylogeny of acariform mites (Acariformes) provides insights on the origin of the four-legged mites (Eriophyoidea), a long branch. *Molecular Phylogenetics and Evolution*. 119, 105–117. https://doi.org/10.1016/j.ympev.2017.10.017

Krivolutsky, D. A., Krasilov, V. A. (1977). Oribatid mites from Upper Jurassic, USSR. In: O. A Skarlato. & S. Balashov (Eds.) (pp. 16–24) *Morphology and Diagnostics of Mites*. Leningrad, Academy of Sciences of the USSR.

Lanfear, R., Calcott, B., Ho, S. Y. W. & Guindon, S. (2012). PartitionFinder: combined selection of partitioning schemes and substitution models for phylogenetic analyses. *Molecular Biology and Evolution*, 29, 1695-1701.

Norton, R. A., Bonamo, P. M., Grierson, J. D., Shear, W. A. (1988). Oribatid mite fossils from a terrestrial Devonian deposit near Gilboa, New York. *Journal of Paleontology*, 62, 259-269.

Pepato, A. R. P. & Klimov, P. B. (2015). Origin and higher-level diversification of acariform mites – evidence from nuclear ribosomal genes, extensive taxon sampling, and secondary structure alignment. *BMC Evolutionary Biology*, 15:178. DOI 10.1186/s12862-015-0458-2.

Pepato, A.R., Vidigal, T.H.D.A. & Klimov, P.B. (2018). Molecular phylogeny of marine mites (Acariformes: Halacaridae), the oldest radiation of extant secondarily marine animals. *Molecular Phylogenetics and Evolution*, 10.1016/j.ympev.2018.08.012

Rambaut, A., Drummond, A. J., Xie, D., Baele, G. & Suchard, M. A. (2018). Posterior summarisation in Bayesian phylogenetics using Tracer 1.7. *Systematic Biology*. syy032. doi:10.1093/sysbio/syy032

Sidorchuk, E. A. & Khaustov, A. A. (2018). A parasite without host: The first fossil pterygosomatid mite (Acari: Prostigmata: Pterygosomatidae) from French Lower Cretaceous amber. *Cretaceous Research*, 91, 131-139.

Sivhed, U. & Wallwork, J. A. (1978). An Early Jurassic oribatid mite from southern Sweden. *Geologiska Foreningens i Stockholm Forhandlingar*, 100, 65-70.

Subías, L. S. & Arillo, A. (2002). Oribatid fossil mites from the Upper Devonian of South Mountain, New York and the Lower Carboniferous of County Antrim, North Ireland (Acariformes, Oribatida). *Estudios del Museo de Ciencias Naturales de Alava*, 17, 93-106.

Wolfe, J. M., Daley, A. C., Legg, D. A. & Edgecombe, G. D. (2016). Fossil calibrations for the arthropod Tree of Life. *Earth-Science Reviews*, 160, 43-110.

Xia, X. (2017). DAMBE6: New tools for microbial genomics, phylogenetics and molecular evolution. *Journal of Heredity*, 108(4), 431-437.

Table S1. Terminals, their taxonomic classification, and GenBank accession numbers for the nuclear ribosomal genes SSU (18S), LSU (28S) and mitochondrial locus COI. This dataset was used to infer our acariform mite time tree (Fig. 2S).

Taxon<br>Order Solifugae	Family or Subfamily	SSU	LSU	COI
Mummucia	Mummucidae	KY922096	KY921963	KY922346
Gluvia dorsalis	Daesiidae	AF007103	KM100936	KM100979
Eremobates	Eremobatidae	AY859573	AY859572	-----
Order Acariformes<br>Endeostigmata				
Nanorchestes	Nanorchestidae	KY922106	KY921973	KY922356
Bimichaelia	Alycidae	KY922112	KY921980	KY922358
Pachygnathus	Alycidae	KY922115	KY921983	KY922360
Alycus aff roseus	Alycidae	KY922116	KY921984	KY922361
Cunliffea	Nematalycidae	KY922118	KY921987	KY922363
Gordialycus	Nematalycidae	KY922131	KY921999	KY922376
Alicorhagia	Alicorhagiidae	JQ000032	JQ000339	KY922381
Micropsammus	Micropsammidae	KY922132	KY922000	KY922377
Terpnacarus gibbosus	Terpnacaridae	KY922140	KY922008	KY922387
Oehserchestidae	Oehserchestidae	KY922143	KY922011	KY922390
Hybalicus	Lordalychidae	KY922103	KY921970	KY922353
Oribatida<br>Palaeosomata				
Palaeacarus kamenskii	Palaeacaridae	JQ000034	JQ000341	KY922451
Beklemishevia galeodula	Ctenacaridae	KP325051	KP325013	----
Enarthronota				
Liochthonius sp. *brevis*-complex	Brachychthoniidae	JQ000035	JQ000342	KY922452
Brachychthoniidae sp	Brachychthoniidae	JQ000036	JQ000343	KY922453
Paralycus	Pediculochelidae	KY922209	KY922080	KY922457
Cosmochthonius lanatus	Cosmochthoniidae	JQ000040	JQ000348	----
Hypochthonius luteus	Hypochthoniidae	JQ000038	JQ000345	KY922455
Haplochthonius simplex	Haplochthoniidae	KY922210	JQ000347	KY922458
Mixonomata				
Euphthiracarus pulchrus	Euphthiracaridae	JQ000041	JQ000349	KY922460
Meristacarus sp.	Lohmaniidae	KP276478	KP276398	----
Epilohmannia pallida	Epilohmanniidae	KY922212	KY922082	KY922461
Parhyposomata				
Gehypochthonius	Gehypochthoniidae	JQ000042	JQ000350	KY922462
Holosomata				
Nothrus borussicus	Nothridae	KY922216	KY922086	KY922468
Camisia segnis	Camisiidae	JQ000043	JQ000351	KY922463
Camisia sp.	Camisiidae	MK014972	MK014995	MK015019
Masthermannia sp	Nanhermanniidae	KY922217	KY922087	----
Afronothrus sp.	Trhypochthoniidae	JQ000045	JQ000353	KY922467
Trhypochthonius americanus	Trhypochthoniidae	JQ000046	JQ000354	KY922469
Brachypylina				
Trachyoribates ovulum	Haplozetidae	HM070342	KP276400	-----
Gymnodamaeus bicostatus	Gymnodamaeidae	MK014973	MK014996	MK015020
Chamobates sp.	Chamobatidae	MK014974	MK014997	MK015021
Zetorchestes micronychus	Zetorchestidae	MK014975	MK014998	MK015022
Pergalumna cf. nervosa	Galumnidae	MK014976	MK014999	MK015023

Neoribates aurantiacus	Parakalummidae	MK014977	MK015000	MK015024
Cultroribula bicultrata	Astegistidae	MK014978	MK015001	MK015025
Siculobata leontonycha	Hemileiidae	MK014979	MK015002	MK015026
Aeroppia sp.	Oppiidae	HM070344	KP276401	-----
Carabodes sp.	Carabodidae	MK014980	MK015003	MK015027
Odontocepheus oblongus	Carabodidae	KY922219	KY922089	KY922471
Cubabodes aff *verrucatus*	Carabodidae	KY922220	KY922090	KY922472
Charassobates aff *tuberosus*	Charassobatidae	KY922221	KY922091	KY922473
Anachipteria howardi	Achipteriidae	JQ000048	JQ000356	-----
Pseudotocepheus amonstruosus	Tetracondylidae	HM070341	KP276403	-----
Astigmata "Acaridia"				
Naiadacarus arboricola	Acaridae	JQ000114	JQ000422	KY922487
Canestrinia pentodontis	Canestriniidae	JQ000084	JQ000392	-----
Dermacarus tamiasciuri	Glycyphagidae	KP325070	KP325032	-----
Nanacarus	Hemisarcoptidae	JQ000068	JQ000376	KY922477
Bonomoia sp	Histiostomatidae	JQ000050	JQ000358	KY922474
Picidectes chapini	Hypoderatidae	JQ000126	JQ000434	-----
Psoroptidia				
Avenzoaria calidridis	Avenzoariidae	KP325068	KP325030	KU203080
Dermatophagoides farinae	Pyroglyphidae	JQ000247	JQ000555	GQ465336
Chirnyssoides amazonae	Sarcoptidae	KP325067	KP325029	-----
Amerodectes aff atyeoi	Proctophyllodidae	KP325069	KP325031	KU203226
Prostigmata Eupodina				
Labidostomma (*Atyeonella*) sp.	Labidostommatidae	KP276485	KP276409	-----
Labidostomatidae gen. sp.	Labidostomatidae	KY922146	KY922014	KY922392
Scolotydeus uralensis	Paratydeidae	MK014981	MK015004	MK015028
Tanytydeus sp.	Paratydeidae	KY922147	KY922015	KY922393
Ereynetidae gen. sp.	Ereynetidae	KY922152	KY922020	KY922398
Tydeidae gen. sp.	Tydeidae	KY922148	KY922016	KY922394
Pseudolorrya sp.	Tydeidae	MK014982	MK015005	MK015029
Tydeus sp.	Tydeidae	MK014983	MK015006	MK015030
Cyta sp.	Bdellidae	MK014985	MK015008	----
Bdella longicornis	Bdellidae	KY922154	KY922023	KY922401
Parabonzia sp.	Cunaxidae	KY922155	KY922024	KY922402
Benoinyssus aff serratus	Eupodidae	KY922150	KY922018	KY922396
Linopodes sp.	Eupodidae	KY922153	KY922022	KY922400
Eupodidae gen. sp.	Eupodidae	MK014984	MK015007	MK015031
Rhagidiidae gen. sp.	Rhagidiidae	KY922151	KY922019	----
Penthalodes sp.	Penthalodidae	KP276486	KP276410	----
Stereotydeus sp	Penthalodidae	KP276487	KP276411	----
Eleutherengona				
Eustigmaeus sp.	Stigmaeidae	KY922175	KY922046	KY922420
Homocaligus sp.	Homocaligidae	KY922176	KY922047	KY922421

Caenolestomyobia faini	Myobiidae	KY922177	KY922048	KY922422
Radfordia elegantula	Myobiidae	KY922178	KY922049	KY922423
Harpyrhynchoides zumpti	Harpirhynchidae	KY922181	KY922052	KY922426
Harpypalpus holopus	Harpirhynchidae	KY922185	KY922056	KY922429
Psorergates sp.	Psorergatidae	KY922186	KY922057	KY922430
Demodex folliculorum	Demodecidae	KY922187	KY922058	KY922431
Barbutia sp.	Barbutiidae	KY922188	KY922059	KY922432
Cyclurobia sp.	Pterygosomatidae	KY922190	KY922061	KY922434
Caligonellidae gen. sp.	Caligonellidae	KY922191	KY922062	KY922435
Raphignathus sp.	Raphignathidae	KY922192	KY922063	KY922436
Favognathus sp.	Cryptognathidae	KY922193	KY922064	KY922437
Linotetranidae gen. sp.	Linotetranidae	KY922194	KY922065	KY922438
Eotetranychus sp.	Tetranychidae	KY922195	KY922066	KY922439
Tetranychus urticae	Tetranychidae	KY922197	KY922068	KY922441
Tenuipalpus sp.	Tenuipalpidae	KY922198	KY922069	KY922442
Brevipalpus sp.	Tenuipalpidae	KY922199	KY922070	KY922443
Neophyllobius sp.	Camerobiidae	KY922200	KY922071	KY922444
Bakericheyla chanayi	Cheyletidae	KY922201	KY922072	KY922445
Chelacheles sp.	Cheyletidae	KY922202	KY922073	KY922446
Cheyletidae gen. sp.	Cheyletidae	KY922203	KY922074	KY922447
Oudemansicheyla sp.	Cheyletidae	HM070362	KP276422	-----
Charadriphilus gallinago	Syringophilidae	KY922205	KY922076	KY922448
Anystina				
Andocaeculus sp.	Caeculidae	KP276488	KP276412	-----
Erythracarus sp.	Anystidae	KP276489	KP276413	-----
Anystis sp.	Anystidae	KY922145	KP325014	KY922391
Adamystidae gen. sp.	Adamystidae	KY922156	KY922026	KY922403
Eutrombicula splendens	Trombiculidae	KY922159	KY922031	MK015032
Acomatacarus arizonensis	Leeuwenhoekiidae	KY922157	KY922029	KY922406
Leptus sp.	Erythraeidae	KP276490	KP276414	-----
Caeculisoma sp.	Erythraeidae	KP276491	KP276415	-----
Balaustium sp.	Erythraeidae	KY922158	KY922030	KY922407
Lasioerythraeus sp.	Erythraeidae	KM100884	KM100950	KM100991
Smaris sp.	Smarididae	KM100885	KM100951	KM100990
Calyptostoma sp.	Calyptostomatidae	KM100878	KM100948	KM100992
Calyptostoma velutinus	Calyptostomatidae	KM100880	KM100949	KM100993
Diplothrombium sp.	Johnstonianidae	KM100930	KM100940	KM100986
Allothrombidium sp.	Trombidiidae	KP276493	KP276417	----
Dactylothrombium pulcherrimum	Microtrombidiidae	GQ864281	KM100939	KM100985
Valgoperuvia paradoxa	Microtrombidiidae	KM100934	KM100943	KM100988
Stygothrombium sp.	Stygothrombidiidae	KM100927	KM100938	KM100995
Arrenurus (A.) sp.	Arrenuridae	KM100875	KM100944	KM101003
Horreolanus orphanus	Bogatiidae	AY620907	KM100946	KM101004
Mideopsis roztoczensis	Mideopsidae	JN018219	JN018316	JN018102
Eylais sp	Eylaidae	KM100887	KM100955	AY393896
Limnochares americana	Limnocharidae	KM100888	KM100956	KM100998
Hydrachna conjecta	Hydrachnidae	JN018220	JN018317	JN018103
Hydrovolzia placophora	Hydrovolziidae	KM100889	KM100957	KM100996

Hydryphantes waynensis	Hydryphantidae	KM100893	KM100959	KM101012
Wandesia sp.	Hydryphantidae	KM100897	KM100960	KM101010
Feltria sp.	Feltriidae	KM100905	KM100965	KM101021
Coaustraliobates cf. cortipes	Hygrobatidae	KM100904	KM100964	KM101024
Limnesia (Limnesiella) marshallae	Limnesiidae	KM100910	KM100969	KM101029
Australotiphys barmutai	Pionidae	KM100903	KM100963	KM101017
Litarachna communis	Pontarachnidae	KM100911	KM100970	KM101025
Unionicola crassipes	Unionicolidae	KM100918	KM100973	KM101023
Frontipoda sp.	Oxidae	KM100919	KM100975	KM101000
Sperchonopsis phreaticus	Sperchontidae	KM100922	KM100978	KM100999
Teutonia cometes	Teutoniidae	JN018224	JN018321	JN018107
Torrenticola amplexa	Torrenticolidae	JN018226	JN018323	JN018191
Halacaroides antoniazae.	Halacarinae	MG751443	MG751417	MG696228
Acarothrix sp.	Copidognathinae	KP276481	KP276405	MG696250
Copidognathus sp1	Copidognathinae	MG751444	MG751418	MG696229
Copidognathus sp2	Copidognathinae	MG751445	MG751419	MG696230
Copidognathus sp3	Copidognathinae	MG751446	MG751420	MG696231
Copidognathus sp.5	Copidognathinae	MK014986	MK015009	----
Agauopsis legionium	Halacarinae	MG751447	MG751421	MG696232
Agauopsis bilophus	Halacarinae	MG751448	MG751422	MG696233
Agauopsis sp.	Halacarinae	MK014987	MK015010	MK015033
Halacarellus sp.	Halacarinae	MG751449	MG751423	MG696234
Halacaropsis cf *hirsuta*	Halacarinae	MG751450	MG751424	MG696235
Halacarus omului	Halacarinae	MG751451	MG751425	MG696236
Thalassarachna sp.	Halacarinae	MG751452	MG751426	MG696237
Agaue sp.	Halixodinae	MG751453	MG751427	MG696238
Bradyagaue sp.	Halixodinae	MG751454	MG751428	MG696239
Limnohalacarus cultellatus	Limnohalacarinae	MG751455	MG751429	-----
Limnohalacarus mamillatus.	Limnohalacarinae	KP276482	KP276406	-----
Scaptognathus sp1	Lohmannellinae	MG751457	MG751431	MG696240
Scaptognathus sp2	Lohmannellinae	MG751458	MG751432	MG696241
Porolohmannella violacea	Porolohmannellinae	MK014988	MK015011	MK015034
Isobactrus setosus	Rhombognathinae	MG751460	MG751434	-----
Isobactrus uniscutatus	Rhombognathinae	MG751461	MG751435	MG696243
Isobactrus sp	Rhombognathinae	MK014989	MK015012	MK015035
Metarhombognathus armatus	Rhombognathinae	KP276483	KP276407	MG696251
Rhombognathus sp.1	Rhombognathinae	MK014990	MK015013	MK015036
Rhombognathus areolatus	Rhombognathinae	MG751463	MG751437	MG696244
Rhombognathus levigatoides (SE)	Rhombognathinae	MG751464	MG751438	MG696245
Rhombognathus levigatoides (NE)	Rhombognathinae	MK014991	MK015014	MK015037
Rhombognathus sp. 2	Rhombognathinae	MK014992	MK015015	MK015038

Rhombognathus sp. 3	Rhombognathinae	MK014993	MK015016	MK015039
Rhombognathus sp. 4	Rhombognathinae	MG751465	MG751439	MG696246
Acaromantis vespucioi	Simognathinae	MG751466	MG751440	MG696247
Simognathus sp1	Simognathinae	MG751467	MG751441	MG696248
Simognathus sp2	Simognathinae	MG751468	MG751442	MG696249
Simognathus sp.3	Simognathinae	MK014994	MK015017	MK015040

Table S2 Fossil calibration points used in BEAST molecular clock phylogenetic analyses. Lognormal prior distribution of ages was set with an offset corresponding to the minimal age and the mean calculated such that 95% of the distribution lies between the minimal ages and the maximum age, with a standard deviation of 1.00. The only exception was the root: its prior distribution was set as an exponential distribution by running preliminary priors-only analyses (Fig. S1) to check whether the prior fossil calibration distributions are affected by priors' interactions. Lineages represent crown groups, unless otherwise indicated.

**Divergence**	**Min. age**	**Max. age**	**Fossil**	**Reference**
**Root**	405.1	514.0	Min.: *Protacarus crani* Max.: *Wisangocaris barbarahardyae*	Wolfe et al., 2016
**Solifugae**	112.6	509.0	Min.: *Cratosolpuga wunderlichi* Max.: *Wisangocaris barbarahardyae*	Wolfe et al., 2016
**Sarcoptiformes**	405.1	509.0	Min.: *Protospeleorchestes pseudoprotacarus*; Max.: *Wisangocaris barbarahardyae*	Dubinin, 1962; Wolfe et al., 2016
**Erythraeoidea**	112.6	410.2	Min.: *Pararainbowia martilli* Max.: *Protacarus crani*	Dunlop, 2007
**Pterygosomatidae**	100.5	410.2	Min.: *Pimeliaphilus* sp. Max.: *Wisangocaris barbarahardyae*	Sidorchuk & Khaustov, 2018
**Cheyletidae**	98.2	410.2	Min.: *Cheyletus burmiticus* Max: *Protacarus crani*	Cockerell, 1917
**stem group Enarthronota**	388.1	509.0	Min.: *Protochthonius gilboa, Devonacarus sellnicki* Máx.: *Wisangocaris barbarahardyae*	Norton et al., 1988

**Lohmanniidae-Hypochthoniidae**	326.4	410.2	Min.: *Palaeohypochthonius jerami*<br>Max.: *Protacarus crani*	Subías & Arillo, 2002
**Brachypilina**	189.8	388.1	Min.: *Hydrozetes* sp.<br>Max.: *Protochthonius gilboa, Devonacarus sellnicki*	Sivhed & Wallwork, 1978
**Trhypochthoniidae**	100.5	388.1	Min.:*Trhypochthonius lopezvallei*<br>Max.: *Protochthonius gilboa, Devonacarus sellnicki*	Arillo et al., 2012
**Astegistidae**	145.0	388.1	Min.: *Cultroribula jurassica*<br>Max.: *Protochthonius gilboa, Devonacarus sellnicki*	Krivolutsky & Krasilov, 1977
**Achipteriidae**	145.0	388.1	Min.: *Achipteria obscura*<br>Max.: *Protochthonius gilboa, Devonacarus sellnicki*	Krivolutsky & Krasilov, 1977

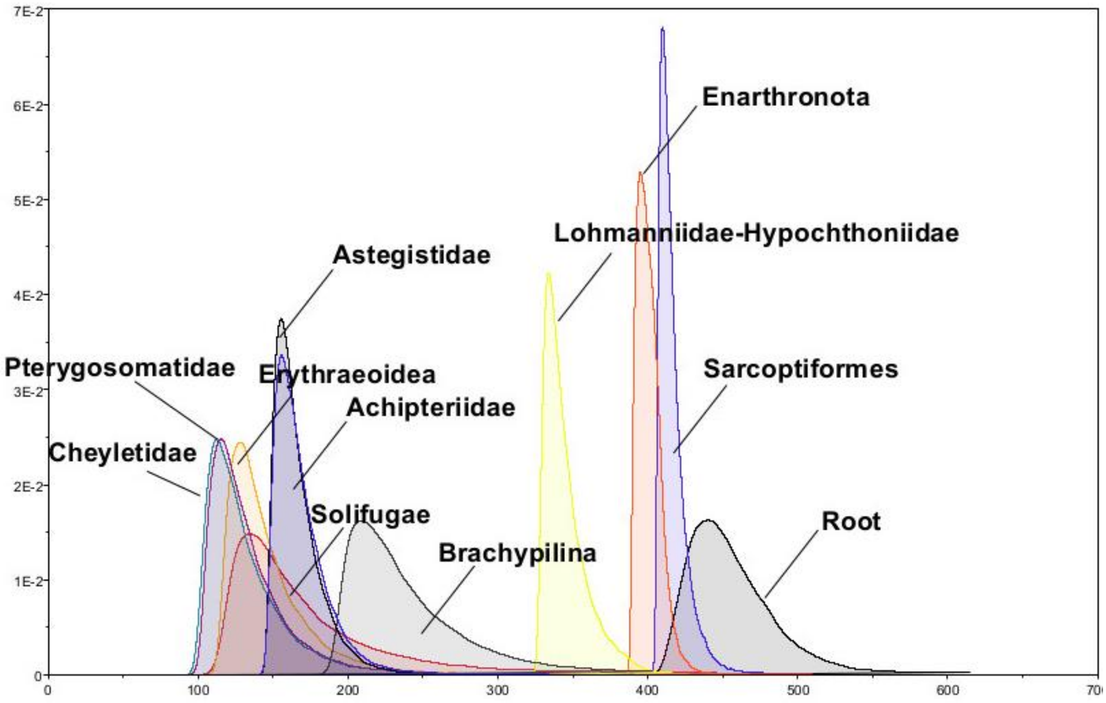

Figure S1. Multiplicative priors on node ages estimated by Tracer. See Table 2 for a list of calibration points and min/max ages. Age distributions represent crown groups, except for Enarthronota (stem group). [label y and x axes]

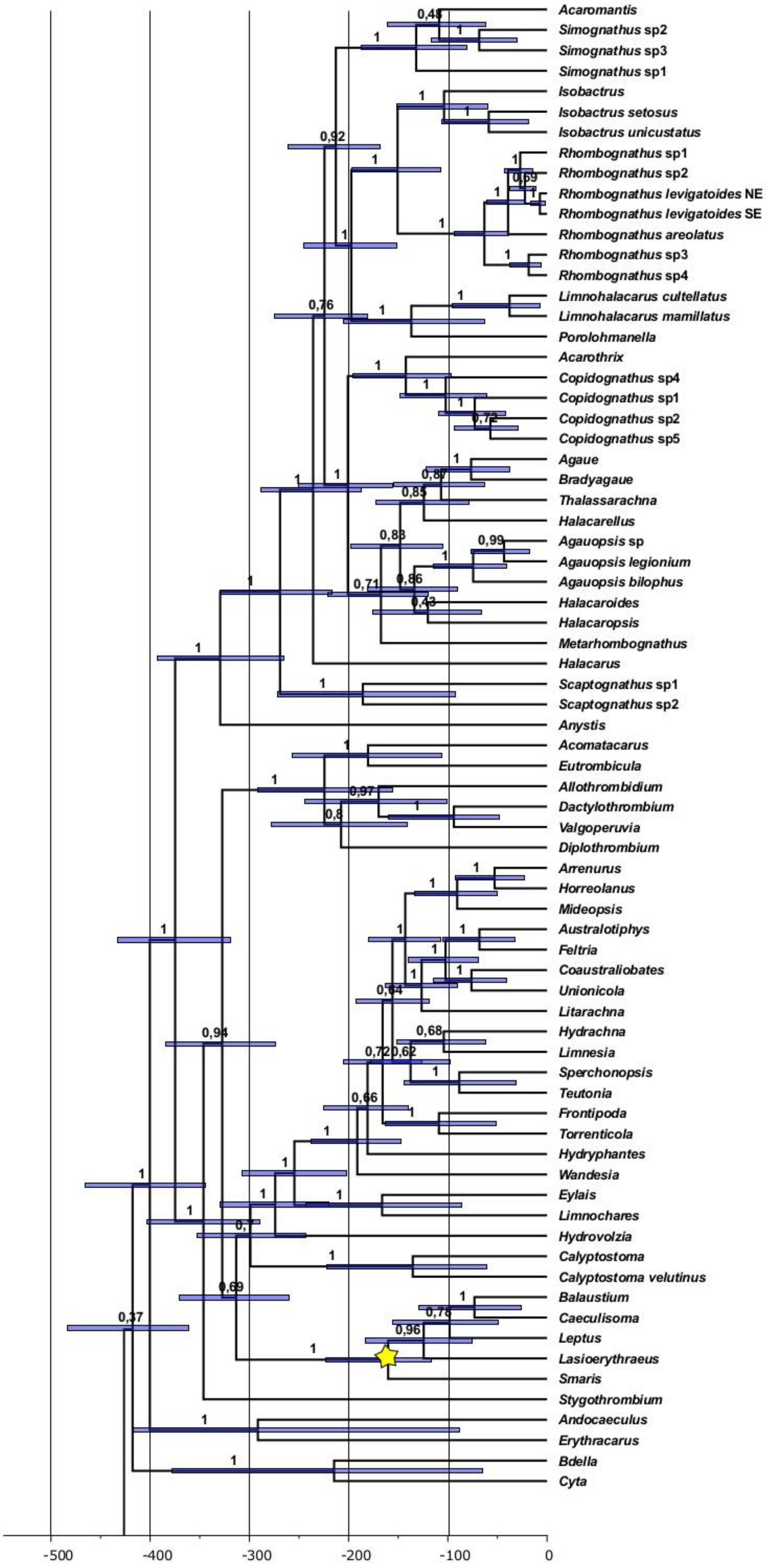

Figure S2A. Time calibrated tree for Acariformes with fossil calibration points indicated by stars. Values above branches are posterior probabilities; bars represent 95% HPD interval.

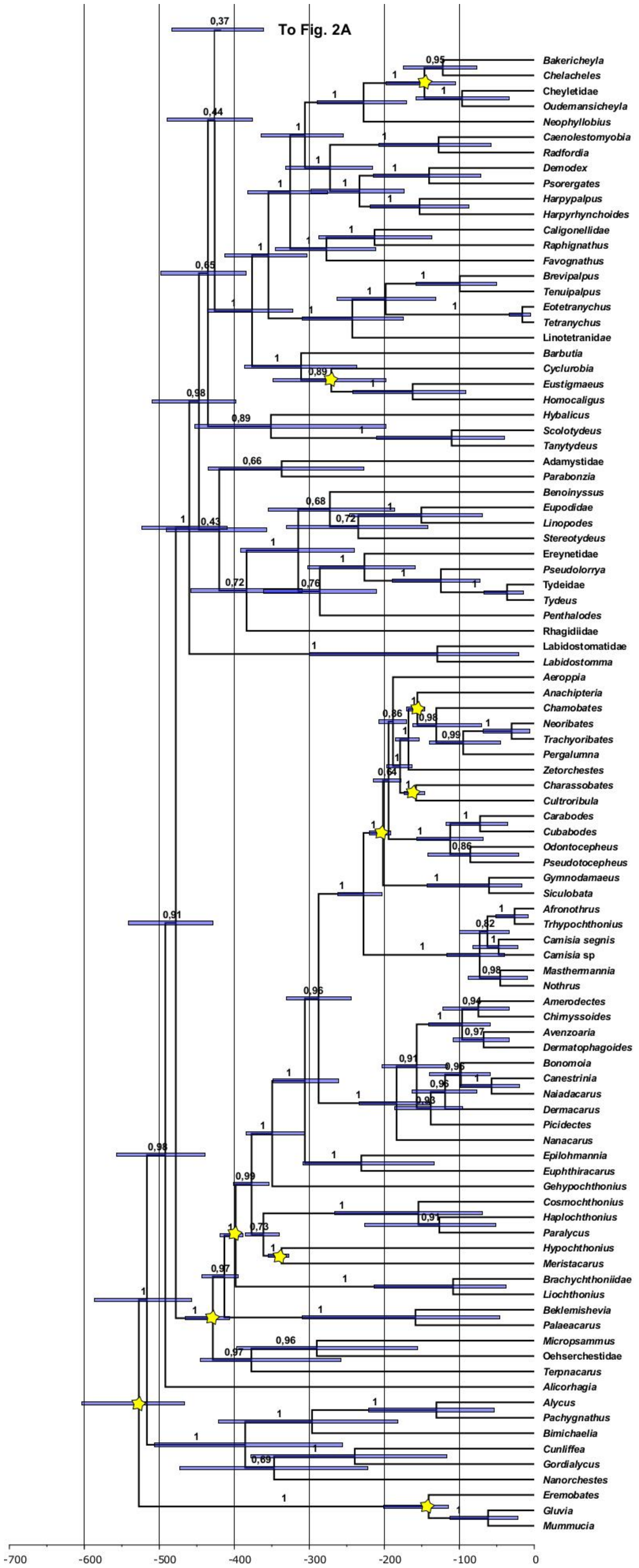

Figure S2B (Continues from Fig. S2A). Time calibrated tree for Acariformes mites with fossil calibration points indicated by stars. Values above branches are posterior probabilities; bars are the 95% HPD interval.
