# Supplementary material for: Evaluating the boundaries of marine biogeographic regions of the Southwestern Atlantic using halacarid mites (Halacaridae), meiobenthic organisms with a low dispersal potential
Source: Ecol Evol. 2019 Nov 7;9(23):13359–74. doi: 10.1002/ece3.5791 (PMC6912894; doi:10.1002/ece3.5791)

Agauopsis legionium

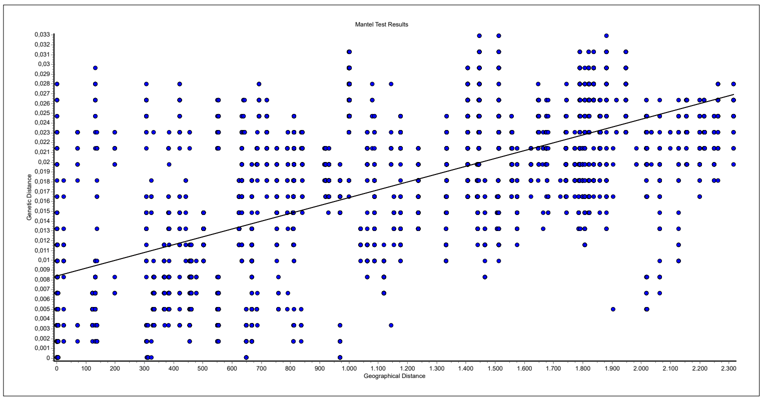

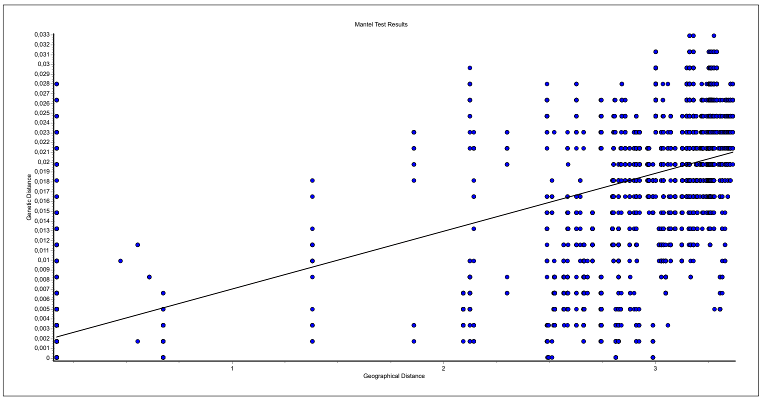

Rhombognathus levigatoides

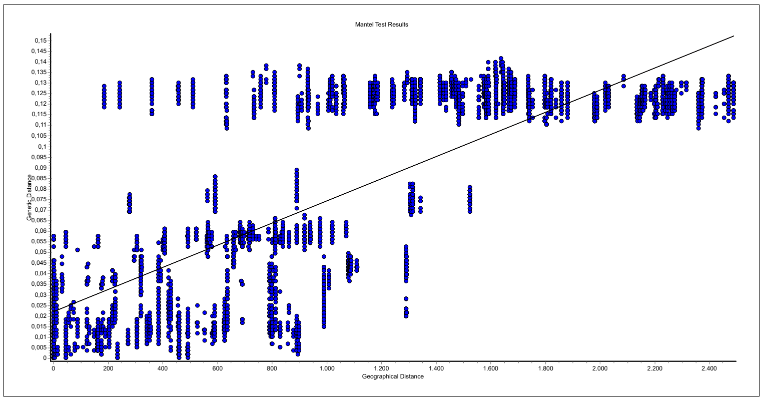

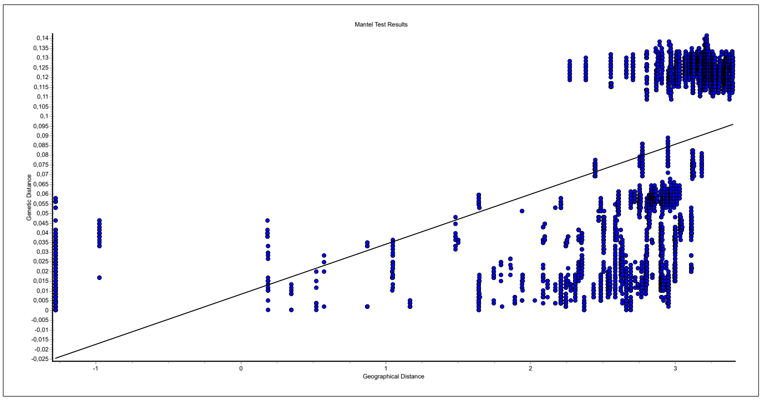

Rhombognathus levigatoides (NE)

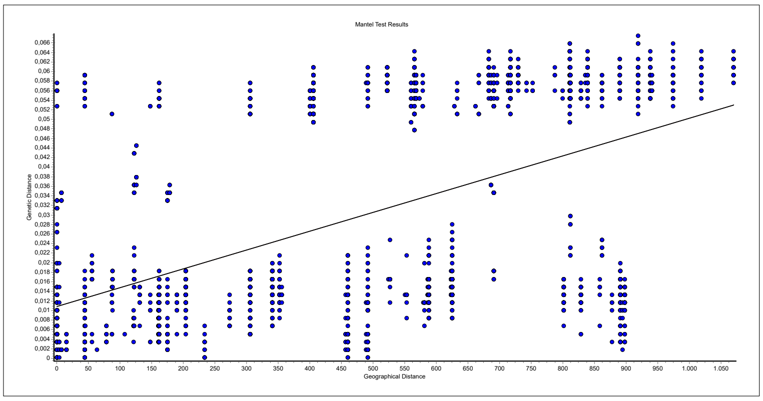

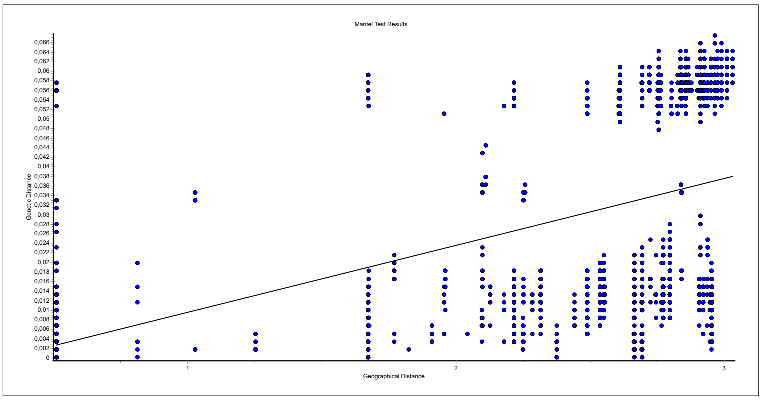

Rhombognathus levigatoides (SE)

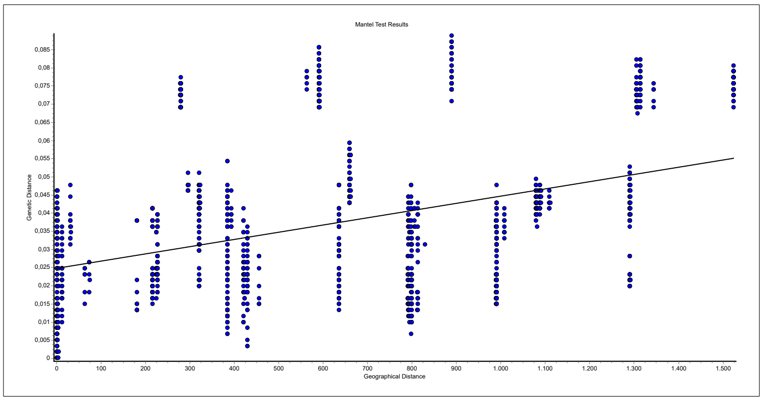

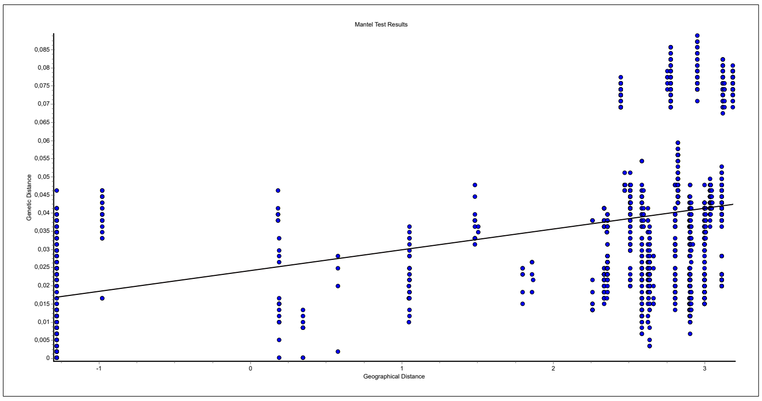

Supplement: Supplementary file 2 [file ECE3-9-13359-s002.pdf]
