# Supplementary material for: Evaluating the boundaries of marine biogeographic regions of the Southwestern Atlantic using halacarid mites (Halacaridae), meiobenthic organisms with a low dispersal potential
Source: Ecol Evol. 2019 Nov 7;9(23):13359–74. doi: 10.1002/ece3.5791 (PMC6912894; doi:10.1002/ece3.5791)
Supplement: Supplementary file 3 [file ECE3-9-13359-s003.docx]

**Appendix 1. Population and samples characterization**

*DNA amplification and* *sequencing*

Specimens were preserved in 95-100 % ethanol and stored at −20 or −80°C. Genomic DNA was extracted from single specimens using a QIAamp DNA Micro kit (Qiagen) following the manufacturer’s protocol except by using two steps of the final elution, leading to a final volume of 70 µl.

The amplification of COI was conducted using nested PCR with parent PCR product being produced employing forward primer COX1_16F (TGANTWTTTTCHACWAAYCAYAA) or alternatively COX1_220F (ATAATHGGDGGDTTYGGIAA) and the reverse primer COX1_1324R (CDGWRTAHCGDCGDGGTAT). The second PCR was performed with primers including M13 forward and reverse tails, respectively (not shown on the sequences): COX1_270F_T (TGAYATRGCNTWYCCICG) COX1 917RT (DGTRAARTADGCHCGDGTRTC). Both rounds of amplifications were performed in 20 µl of final volume, with Platinum Taq DNA Polymerase (Invitrogen) in a Mastercycler gradient, Eppendorf thermocycler. Master mix for initial PCR contained 2.0 µl of PCR buffer (1X), 1.4 µl MgSO4 (50 mM), 1.4 µL of dNTP (10 mM each), 0.8 µl of each oligonucleotide primer. The first round of amplification used 1-3 µl of genomic DNA and included an initial denaturing step of 4 min at 94°C, ten amplification cycles, comprising: denaturing at 94°C for 30s, annealing at 48°C for 35s, and extension at 72°C for 2 min, 18 amplification cycles changing the annealing temperature to 51°C, and a final step of extension of 5 min at 72°C. The second round employed 0.5 µl from the parent PCR product and included an initial denaturing step of 4 min at 94°C, 35 amplification cycles, comprising: denaturing at 94°C for 30s, annealing at 45°C for 60 s, and extension at 72°C for 2 min, and a final step of extension of 5 min. at 72°C. Amplification protocols for other loci are given in Pepato et al. (2018).

PCR products were purified using the Ampure® (Agencourt) kit and sequenced using a 3730 DNA Analyzer. M13 FORW/REV primers were used for sequencing. Chromatograms were resolved in ChromasPro 1.41 (Technelysium Pty Ltd).

**Results**

*Agauopsis legionium*

Eighty-nine sequenced individuals formed 11 populations distributed from the Amazonian region of Pará State in the north (0°36’S 47°20’W) to Santa Catarina State in the south (26°48’ S 48°35’W) (Table S1, Fig. 1). The total alignment length was 609 nucleotide positions, of which 70 sites were variable and 46 parsimony informative; after amino acid translation, 12 amino acid positions were variable, and six were parsimony informative. The data set presented 58 different haplotypes and a haplotype diversity Hd = 0,9816. The nucleotide diversity, π = 10.95 ±5.6.

*Rhombognathus levigatoides*

One hundred twenty eight sequenced individuals were grouped in 14 populations, ranging from the Parnaíba River Delta, Piaui State to Torres, Rio Grande do Sul State (2° 48' S to 29° 21'S). Out of 609 nucleotide alignment positions, 150 were variable and 132 were parsimony informative; 13 amino acid residues were variable, and nine of them were parsimony informative. There were 99 distinct haplotypes and with Haplotype diversity Hd = 0.9940; the nucleotide diversity was π = 52.21±25.2 (Table S1).

**References**

Pepato, A. R., Vidigal, T.H.D.A. & Klimov, P.B. (2018). Molecular phylogeny of marine mites (Acariformes: Halacaridae), the oldest radiation of extant secondarily marine animals. *Molecular Phylogenetics and Evolution*, 10.1016/j.ympev.2018.08.012

**Table S1**. Sequenced individuals of *Rhombognathus levigatoides* Pepato & Rocha, 2007 complex and *Agauopsis legionium* Pepato & Tiago, 2005. Provinces and ecoregions of the Brazilian coast follow Spalding et al. (2007). When applicable, nucleotide (π) and haplotypic (Hd) diversities are given as mean ± SD.

| **Province** | **Ecoregion** | **#Locality** | **Latitude** | **Longitude** | | ***R. levigatoides*** | ***A. legionium*** | **Pop.** | ***R. levigatoides*** | ***A. legionium*** |
| --- | --- | --- | --- | --- | --- | --- | --- | --- | --- | --- |
| North Brazil Shelf | Amazonia | 1. PA | -0.596325 | | -47.330775 | -- | 16 | N01 | NA | π= 0.00321±0.00082;  Hd= 0.831± 0.085 |
|  |  | 1. MA | -2.490997 | | -44.306447 | **--** | 01 |  |  |  |
| Tropical Southwestern Atlantic | Northeastern Brazil | 1. PI | -2.803239 | | -41.729286 | 05 | -- | NE1 | π=0.00493±0.00150;  Hd=1.000±0.01600 | NA |
|  |  | 1. PI | -2.922886 | | -41.344211 | **--** | 01 | NE2 | π=0.01026±0.00289;  Hd=0.972±0.064 | π=0.00239±0.00071;  Hd= 0.727±0.144 |
|  |  | 1. CE | -3.688481 | | -38.611197 | 01 | 04 |  |  |  |
|  |  | 1. CE | -3.686830 | | -38.640300 | 08 | 06 |  |  |  |
|  |  | 1. RN | -6.226086 | | -35.044461 | 09 | 06 | NE3 | π=0.00607±0.00159;  Hd=0.944±0.070 | π=0.00372±0.00143;  Hd=0.867±0.129 |
|  |  | 1. PB | -6.686881 | | -34.930664 | 08 | -- | NE4 | π=0.02673±0.00749;  Hd=0.944±0.070 | NA |
|  |  | 1. PB | -7.145014 | | -34.806472 | 01 | -- |  |  |  |
|  |  | 1. PE | -7.630539 | | -34.808539 | 01 | 07 | NE5 | π=0.00339± 0.00081;  Hd=0.867±0.107 | π= 0.01345±0.00419;  Hd=0.952±0.096 |
|  |  | 1. PE | -8.457536 | | -34.982994 | 08 | -- |  |  |  |
|  |  | 1. PE | -8.302775 | | -34.947014 | 01 | -- |  |  |  |
|  |  | 1. AL | -9.840211 | | -35.889522 | 12 | -- | NE6 | π=0.00440±0.00095;  Hd=0.909±0.079 | NA |
|  | Eastern Brazil | 1. BA | -13.00728 | | -38.454197 | 02 | -- | E01 | π=0.01943±0.00306;  Hd=0.861±0.087 | π=0.00361±0.00090;  Hd=0.933±0.122 |
|  |  | 1. BA | -13.01021 | | -38.520697 | 06 | 06 |  |  |  |
|  |  | 1. BA | -14.28396 | | -38.984781 | 04 | 05 | E02 | π=0.01595±0.00266;  Hd=1.000±0.076 | π=0.00164±0.00057;  Hd=0.700± 0.218 |
|  |  | 1. BA | -14.92399 | | -39.017847 | 03 | -- |  |  |  |
|  |  | 1. BA | -17.063500 | | -39.170389 | 05 | -- | E03 | π=0.01018±0.00181;  Hd=1.000±0.126 | NA |
|  |  | 1. ES | -19.93689 | | -40.132667 | -- | 10 | E04 | π=0.00775±0.00122;  Hd=0.889±0.091 | π=0.00234±0.00054;  Hd=0.844±0.103 |
|  |  | 1. ES | -20.031994 | | -40.159044 | 01 | -- |  |  |  |
|  |  | 1. ES | -20.038350 | | -40.177894 | 08 | -- |  |  |  |
| Warm Temperate Southwestern Atlantic | Southeastern Brazil | 1. RJ | -22.598858 | | -41.990372 | 01 | -- | SE1 | π=0.02716±0.00391;  Hd=0.956±0.045 | π=0.00788±0.00150;  Hd=0.900±0.161 |
|  |  | 1. RJ | -22.96143 | | -42.020378 | 03 | 01 |  |  |  |
|  |  | 1. RJ | -22.96414 | | -42.012556 | 08 | -- |  |  |  |
|  |  | 1. RJ | -22.97928 | | -42.019367 | 02 | 01 |  |  |  |
|  |  | 1. RJ | -22.98149 | | -42.038006 | -- | 03 |  |  |  |
|  |  | 1. SP | -23.57601 | | -45.308014 | -- | 02 | SE2 | π=0.01861±0.00431;  Hd=0.952±0.096 | π= 0.01235±0.00297;  Hd=0.905±0.103 |
|  |  | 1. SP | -23.82197 | | -45.410717 | 07 | 09 |  |  |  |
|  |  | 1. SP | -25.20194 | | -47.984406 | -- | 04 | SE3 | NA | π=0.01423± 0.00356;  Hd= 1.000± 0.177 |
|  |  | 1. SC | -26.774389 | | -48,636583 | 01 | -- | SE4 | π=0.02015±0.00196;  Hd=1.000±0.034 | π= 0.01314±0.00245  Hd=0.905±0.01067 |
|  |  | 1. SC | -26,780750 | | -48,603583 | 04 | -- |  |  |  |
|  |  | 1. SC | -26.80260 | | -48.596133 | -- | 04 |  |  |  |
|  |  | 1. SC | -26,924150 | | -48,634781 | 05 | -- |  |  |  |
|  |  | 1. SC | -27.74820 | | -48.499236 | 01 | 03 |  |  |  |
|  | Rio Grande | 1. RS | -29.358381 | -49.733639 | | 11 | --- | RG1 | π=0.02365±0.00348;  Hd=0.891±0.074 | NA |

**Table S2.** GenBank accession numbers and vouchering of newly sequenced COI for individuals belonging to four morphospecies of the genera *Agauopsis* and *Rhombognathus*.

| Vouchering (UFMG-AC) | Locality | Coordinates (Lat./Long) | GenBank accession |
| --- | --- | --- | --- |
| *Agauopsis bilophus* |  |  |  |
| 1301135 | Pedra do Xáreu, Cabo de Santo Agostinho, PE, 10.VIII.2007 | -8.30492778,-34.9482611 | MH999696 |
| 1301136 | Pedra do Xáreu, Cabo de Santo Agostinho, PE, 10.VIII.2007 | -8.30492778,-34.9482611 | MH999697 |
| 1700728 | Ponta de Pedra, Pituba, Salvador, BA, 20/X/2015 | -13.007275,-38.45419722 | MH999698 |
| 1700729 | Ponta de Pedra, Pituba, Salvador, BA, 20/X/2015 | -13.007275,-38.45419722 | MH999699 |
| 1700730 | Ponta de Pedra, Pituba, Salvador, BA, 20/X/2015 | -13.007275,-38.45419722 | MH999700 |
| 1703950 | Praia do Resende, Itacaré, BA, 30/X/2015 | -14.283958, -38.984781 | MH999701 |
| 1704013 | Praia do Resende, Itacaré, BA, 30/X/2015 | -14.283958, -38.984781 | MH999702 |
| 1300477 | Enseada das Garças, Fundão, ES, 10.VIII.2014 | -20.0319944,-40.1590444 | MH999703 |
| 1301111 | Praia do Forno, Arraial do Cabo, RJ, 19.III.2015 | -22.964136, -42.012556 | MH999704 |
| 1301144 | Ilha de Cabo Frio, Arraial do Cabo, RJ, 18.III.2015 | -22.9986889,-42.0031694 | MH999705 |
| 1301145 | Ilha de Cabo Frio, Arraial do Cabo, RJ, 18.III.2015 | -22.9986889,-42.0031694 | MH999706 |
| 1301142 | Ilha de Cabo Frio, Arraial do Cabo, RJ, 18.III.2015 | -22.9986889,-42.0031694 | MH999707 |
| 1301160 | Prainha, Arraial do Cabo, RJ, 16.III.2015 | -22.961428, -42.020378 | MH999709 |
| 1301149 | Praia das Cabeçudas, Itajaí, SC, 06.II.2015 | -26,924150, -48,634781 | MH999708 |
| 1300506 | Praia do Quilombo, Penha, SC, 05.II.2015 | -26.774389, -48,6365833 | MH999710 |
| 1301148 | Praia das Cabeçudas, Itajaí, SC, 06.II.2015 | -26,924150, -48,634781 | MH999711 |
| *Agauopsis legionium* |  |  |  |
| 1300487 | Praia do Farol Velho, Salinópolis, PA, 09.VII. 2014 | -0.596325, -47.330775 | MH999712 |
| 1300488 | Praia do Farol Velho, Salinópolis, PA, 09.VII. 2014 | -0.596325, -47.330775 | MH999713 |
| 1300496 | Praia do Farol Velho, Salinópolis, PA, 09.VII. 2014 | -0.596325, -47.330775 | MH999714 |
| 1300497 | Praia do Farol Velho, Salinópolis, PA, 09.VII. 2014 | -0.596325, -47.330775 | MH999715 |
| 1301128 | Praia do Farol Velho, Salinópolis, PA, 09.VII. 2014 | -0.596325, -47.330775 | MH999716 |
| 1301129 | Praia do Farol Velho, Salinópolis, PA, 09.VII. 2014 | -0.596325, -47.330775 | MH999717 |
| 1301130 | Praia do Farol Velho, Salinópolis, PA, 09.VII. 2014 | -0.596325, -47.330775 | MH999718 |
| 1301131 | Praia do Farol Velho, Salinópolis, PA, 09.VII. 2014 | -0.596325, -47.330775 | MH999719 |
| 1301133 | Praia do Farol Velho, Salinópolis, PA, 09.VII. 2014 | -0.596325, -47.330775 | MH999720 |
| 1301162 | Praia do Farol Velho, Salinópolis, PA, 09.VII. 2014 | -0.596325, -47.330775 | MH999721 |
| 1301163 | Praia do Farol Velho, Salinópolis, PA, 09.VII. 2014 | -0.596325, -47.330775 | MH999722 |
| 1301164 | Praia do Farol Velho, Salinópolis, PA, 09.VII. 2014 | -0.596325, -47.330775 | MH999723 |
| 1301165 | Praia do Farol Velho, Salinópolis, PA, 09.VII. 2014 | -0.596325, -47.330775 | MH999724 |
| 1301166 | Praia do Farol Velho, Salinópolis, PA, 09.VII. 2014 | -0.596325, -47.330775 | MH999725 |
| 1301167 | Praia do Farol Velho, Salinópolis, PA, 09.VII. 2014 | -0.596325, -47.330775 | MH999726 |
| 1301168 | Praia do Farol Velho, Salinópolis, PA, 09.VII. 2014 | -0.596325, -47.330775 | MH999727 |
| 1704159 | Ponta de Areia, São Luis, MA, 17.IX.2016 | -2.490997, -44.306447 | MH999728 |
| 1704337 | Cajueiro da Praia, PI, 20.IX.2016 | -2.922886, -41.344211 | MH999729 |
| 1700740 | Praia de Iparaná, Caucaia, CE, 22.IX.2016 | -3.688481, -38.611197 | MH999730 |
| 1700742 | Praia de Iparaná, Caucaia, CE, 22.IX.2016 | -3.688481, -38.611197 | MH999731 |
| 1700745 | Praia de Iparaná, Caucaia, CE, 22.IX.2016 | -3.688481, -38.611197 | MH999732 |
| 1700749 | Praia de Iparaná, Caucaia, CE, 22.IX.2016 | -3.688481, -38.611197 | MH999733 |
| 1704269 | Praia do Pacheco, Caucaia, CE, 21.IX.2016 | -3.686830, -38.640300 | MH999734 |
| 1704270 | Praia do Pacheco, Caucaia, CE, 21.IX.2016 | -3.686830, -38.640300 | MH999735 |
| 1704271 | Praia do Pacheco, Caucaia, CE, 21.IX.2016 | -3.686830, -38.640300 | MH999736 |
| 1704272 | Praia do Pacheco, Caucaia, CE, 21.IX.2016 | -3.686830, -38.640300 | MH999737 |
| 1704273 | Praia do Pacheco, Caucaia, CE, 21.IX.2016 | -3.686830, -38.640300 | MH999738 |
| 1704275 | Praia do Pacheco, Caucaia, CE, 21.IX.2016 | -3.686830, -38.640300 | MH999739 |
| 174039 | Praia de Pipa, Tibau do Sul, RN, 17.II.2017 | -6.226086, -35.044461 | MH999740 |
| 174172 | Praia de Pipa, Tibau do Sul, RN, 17.II.2017 | -6.226086, -35.044461 | MH999745 |
| 174184 | Praia de Pipa, Tibau do Sul, RN, 17.II.2017 | -6.226086, -35.044461 | MH999741 |
| 174185 | Praia de Pipa, Tibau do Sul, RN, 17.II.2017 | -6.226086, -35.044461 | MH999742 |
| 174236 | Praia de Pipa, Tibau do Sul, RN, 17.II.2017 | -6.226086, -35.044461 | MH999743 |
| 174237 | Praia de Pipa, Tibau do Sul, RN, 17.II.2017 | -6.226086, -35.044461 | MH999744 |
| 174031 | Ponta de Pedra, Goiana, PE, 13.II.2017 | -7.630539, -34.808539 | MH999746 |
| 174032 | Ponta de Pedra, Goiana, PE, 13.II.2017 | -7.630539, -34.808539 | MH999747 |
| 1704033 | Ponta de Pedra, Goiana, PE, 13.II.2017 | -7.630539, -34.808539 | MH999748 |
| 1704180 | Ponta de Pedra, Goiana, PE, 13.II.2017 | -7.630539, -34.808539 | MH999749 |
| 1704182 | Ponta de Pedra, Goiana, PE, 13.II.2017 | -7.630539, -34.808539 | MH999750 |
| 1704183 | Ponta de Pedra, Goiana, PE, 13.II.2017 | -7.630539, -34.808539 | MH999751 |
| 1704190 | Ponta de Pedra, Goiana, PE, 13.II.2017 | -7.630539, -34.808539 | MH999752 |
| 1702586 | Barra, Salvador, BA, 16.VIII.2016 | -13.010208, -38.520697 | MH999753 |
| 1702587 | Barra, Salvador, BA, 16.VIII.2016 | -13.010208, -38.520697 | MH999754 |
| 1702588 | Barra, Salvador, BA, 16.VIII.2016 | -13.010208, -38.520697 | MH999755 |
| 1704199 | Barra, Salvador, BA, 16.VIII.2016 | -13.010208, -38.520697 | MH999756 |
| 1704200 | Barra, Salvador, BA, 16.VIII.2016 | -13.010208, -38.520697 | MH999757 |
| 1704201 | Barra, Salvador, BA, 16.VIII.2016 | -13.010208, -38.520697 | MH999758 |
| 1700739 | Praia do Resende, Itacaré BA, 30.X.2015 | -14.283958, -38.984781 | MH999759 |
| 1704018 | Praia do Resende, Itacaré BA, 30.X.2015 | -14.283958, -38.984781 | MH999760 |
| 1704019 | Praia do Resende, Itacaré BA, 30.X.2015 | -14.283958, -38.984781 | MH999761 |
| 1704020 | Praia do Resende, Itacaré BA, 30.X.2015 | -14.283958, -38.984781 | MH999762 |
| 1704021 | Praia do Resende, Itacaré BA, 30.X.2015 | -14.283958, -38.984781 | MH999763 |
| 174001 | Praia do Padre, Aracruz, ES, 08.X.2014 | -19.936889, -40.132667 | MH999764 |
| 174002 | Praia do Padre, Aracruz, ES, 08.X.2014 | -19.936889, -40.132667 | MH999765 |
| 174003 | Praia do Padre, Aracruz, ES, 08.X.2014 | -19.936889, -40.132667 | MH999766 |
| 174004 | Praia do Padre, Aracruz, ES, 08.X.2014 | -19.936889, -40.132667 | MH999767 |
| 174005 | Praia do Padre, Aracruz, ES, 08.X.2014 | -19.936889, -40.132667 | MH999768 |
| 174006 | Praia do Padre, Aracruz, ES, 08.X.2014 | -19.936889, -40.132667 | MH999769 |
| 174223 | Praia do Padre, Aracruz, ES, 08.X.2014 | -19.936889, -40.132667 | MH999770 |
| 174224 | Praia do Padre, Aracruz, ES, 08.X.2014 | -19.936889, -40.132667 | MH999771 |
| 174225 | Praia do Padre, Aracruz, ES, 08.X.2014 | -19.936889, -40.132667 | MH999772 |
| 174226 | Praia do Padre, Aracruz, ES, 08.X.2014 | -19.936889, -40.132667 | MH999773 |
| 1301154 | Ilha dos Franceses, Arraial do Cabo, RJ, 19.III.2015 | -22.981494, -42.038006 | MH999774 |
| 1301159 | Praia dos Anjos, Arraial do Cabo, RJ, 17.III.2015 | -22.979281, -42.019367 | MH999775 |
| 1301161 | Prainha, Arraial do Cabo, RJ, 16.III.2015 | -22.961428, -42.020378 | MH999776 |
| 1704384 | Ilha dos Franceses, Arraial do Cabo, RJ, 19.III.2015 | -22.981494, -42.038006 | MH999777 |
| 1704385 | Ilha dos Franceses, Arraial do Cabo, RJ, 19.III.2015 | -22.981494, -42.038006 | MH999778 |
| 174366 | Praia de Mococa, Caraguatatuba, SP, 07.III.2016 | -23.576014, -45.308014 | MH999779 |
| 174367 | Praia de Mococa, Caraguatatuba, SP, 07.III.2016 | -23.576014, -45.308014 | MH999780 |
| 174386 | Praia Preta, São Sebastião, SP, 09.III.2016 | -23.821972, -45.410717 | MH999781 |
| 174387 | Praia Preta, São Sebastião, SP, 09.III.2016 | -23.821972, -45.410717 | MH999782 |
| 174389 | Praia Preta, São Sebastião, SP, 09.III.2016 | -23.821972, -45.410717 | MH999783 |
| 174390 | Praia Preta, São Sebastião, SP, 09.III.2016 | -23.821972, -45.410717 | MH999784 |
| 174391 | Praia Preta, São Sebastião, SP, 09.III.2016 | -23.821972, -45.410717 | MH999785 |
| 174277 | Praia Preta, São Sebastião, SP, 09.III.2016 | -23.821972, -45.410717 | MH999786 |
| 174278 | Praia Preta, São Sebastião, SP, 09.III.2016 | -23.821972, -45.410717 | MH999787 |
| 174279 | Praia Preta, São Sebastião, SP, 09.III.2016 | -23.821972, -45.410717 | MH999788 |
| 174280 | Praia Preta, São Sebastião, SP, 09.III.2016 | -23.821972, -45.410717 | MH999789 |
| 1301018 | Marujá, Ilha do Cardoso, Cananeia, SP, 02.I.2012 | -25.201944, -47.984406 | MH999790 |
| 1301151 | Marujá, Ilha do Cardoso, Cananeia, SP, 02.I.2012 | -25.201944, -47.984406 | MH999791 |
| 1301152 | Marujá, Ilha do Cardoso, Cananeia, SP, 02.I.2012 | -25.201944, -47.984406 | MH999792 |
| 1301153 | Marujá, Ilha do Cardoso, Cananeia, SP, 02.I.2012 | -25.201944, -47.984406 | MH999793 |
| 1301155 | Praia Vermelha, Penha, SC, 05.II.2015 | -26.802603, -48.596133 | MH999794 |
| 1301156 | Praia Vermelha, Penha, SC, 05.II.2015 | -26.802603, -48.596133 | MH999795 |
| 1301157 | Praia Vermelha, Penha, SC, 05.II.2015 | -26.802603, -48.596133 | MH999796 |
| 1301158 | Praia Vermelha, Penha, SC, 05.II.2015 | -26.802603, -48.596133 | MH999797 |
| 174381 | Praia da Armação, Florianópolis, SC, 07.II.2015 | -27.748200, -48.499236 | MH999798 |
| 174382 | Praia da Armação, Florianópolis, SC, 07.II.2015 | -27.748200, -48.499236 | MH999799 |
| 174383 | Praia da Armação, Florianópolis, SC, 07.II.2015 | -27.748200, -48.499236 | MH999800 |
| *Rhombognathus* *areolatus* |  |  |  |
| 1704174 | Praia das Trincheiras, Baía da Traição, PB, 16/II/2017 | -6.686881,-34.930664 | MH999551 |
| 1704008 | Pedra do Xáreu, Itapoama, PE, 12/II/2017 | -8.302775, -34.947014 | MH999552 |
| 1704178 | Pedra do Xáreu, Itapoama, PE, 12/II/2017 | -8.302775, -34.947014 | MH999553 |
| 1704192 | Praia da Sereia, Maceió, AL, 21/VIII/2016 | -9.566364,-35.645336 | MH999554 |
| 1704193 | Praia da Sereia, Maceió, AL, 21/VIII/2016 | -9.566364,-35.645336 | MH999555 |
| 1703991 | Ponta de Pedra, Pituba, Salvador, BA, 20/X/2015 | -13.007275,-38.454197 | MH999556 |
| 1704197 | Praia do Moreira, Cumuruxatiba, Prado, BA, 5/I/2015 | -17.0635,-39.170389 | MH999557 |
| 1704196A | Praia do Moreira, Cumuruxatiba, Prado, BA, 5/I/2015 | -17.0635,-39.170389 | MH999558 |
| 1704195 | Praia do Moreira, Cumuruxatiba, Prado, BA, 5/I/2015 | -17.0635,-39.170389 | MH999559 |
| 1704196B | Praia do Moreira, Cumuruxatiba, Prado, BA, 5/I/2015 | -17.0635,-39.170389 | MH999560 |
| 1300473 | Enseada das Garças, Fundão, ES, 10.VIII.2014 | -20.0319944,-40.1590444 | MH999561 |
| 1600622 | Enseada das Garças, Fundão, ES, 10.VIII.2014 | -20.0319944,-40.1590444 | MH999562 |
| 1600623 | Enseada das Garças, Fundão, ES, 10.VIII.2014 | -20.0319944,-40.1590444 | MH999563 |
| 1600625 | Enseada das Garças, Fundão, ES, 10.VIII.2014 | -20.0319944,-40.1590444 | MH999564 |
| 1600643 | Enseada das Garças, Fundão, ES, 10.VIII.2014 | -20.0319944,-40.1590444 | MH999565 |
| 1600646 | Enseada das Garças, Fundão, ES, 10.VIII.2014 | -20.0319944,-40.1590444 | MH999566 |
| 1600647 | Enseada das Garças, Fundão, ES, 10.VIII.2014 | -20.0319944,-40.1590444 | MH999567 |
| *Rhombognathus levigatoides* |  |  |  |
| 172573 | Pedra do Sal, Parnaíba, PI, 19.IX.2016 | -2.03853, -41.730247 | MH999569 |
| 174400 | Pedra do Sal, Parnaíba, PI, 19.IX.2016 | -2.03853, -41.730247 | MH999568 |
| 174411 | Pedra do Sal, Parnaíba, PI, 19.IX.2016 | -2.03853, -41.730247 | MH999570 |
| 174304 | Pedra do Sal, Parnaíba, PI, 19.IX.2016 | -2.03853, -41.730247 | MH999571 |
| 174362 | Pedra do Sal, Parnaíba, PI, 19.IX.2016 | -2.03853, -41.730247 | MH999572 |
| 170750 | Praia de Iparaná, Caucaia, CE, 22.IX.2016 | -2.03853, -41.730247 | MH999573 |
| 1704058 | Praia do Pacheco, Caucaia, CE, 21.IX.2016 | -3.686830, -38.640300 | MH999574 |
| 1704221 | Praia do Pacheco, Caucaia, CE, 21.IX.2016 | -3.686830, -38.640300 | MH999575 |
| 1704369A | Praia do Pacheco, Caucaia, CE, 21.IX.2016 | -3.686830, -38.640300 | MH999576 |
| 1704369B | Praia do Pacheco, Caucaia, CE, 21.IX.2016 | -3.686830, -38.640300 | MH999577 |
| 1704369C | Praia do Pacheco, Caucaia, CE, 21.IX.2016 | -3.686830, -38.640300 | MH999578 |
| 1704265 | Praia do Pacheco, Caucaia, CE, 21.IX.2016 | -3.686830, -38.640300 | MH999579 |
| 1704370 | Praia do Pacheco, Caucaia, CE, 21.IX.2016 | -3.686830, -38.640300 | MH999580 |
| 1704267 | Praia do Pacheco, Caucaia, CE, 21.IX.2016 | -3.686830, -38.640300 | MH999581 |
| 1704242 | Praia de Pipa, Tibau do Sul, RN, 17.II.2017 | -6.226086, -35.044461 | MH999582 |
| 1704243 | Praia de Pipa, Tibau do Sul, RN, 17.II.2017 | -6.226086, -35.044461 | MH999583 |
| 1704244 | Praia de Pipa, Tibau do Sul, RN, 17.II.2017 | -6.226086, -35.044461 | MH999584 |
| 1704295 | Praia de Pipa, Tibau do Sul, RN, 17.II.2017 | -6.226086, -35.044461 | MH999585 |
| 1704297 | Praia de Pipa, Tibau do Sul, RN, 17.II.2017 | -6.226086, -35.044461 | MH999586 |
| 1704405 | Praia de Pipa, Tibau do Sul, RN, 17.II.2017 | -6.226086, -35.044461 | MH999587 |
| 1704409 | Praia de Pipa, Tibau do Sul, RN, 17.II.2017 | -6.226086, -35.044461 | MH999588 |
| 1704410 | Praia de Pipa, Tibau do Sul, RN, 17.II.2017 | -6.226086, -35.044461 | MH999589 |
| 1704301 | Praia de Pipa, Tibau do Sul, RN, 17.II.2017 | -6.226086, -35.044461 | MH999590 |
| 1704255 | Arrecife do Farol, Baia da Traição, PB, 16.II.2017 | -6.686881, -34.930664 | MH999591 |
| 1704365 | Arrecife do Farol, Baia da Traição, PB, 16.II.2017 | -6.686881, -34.930664 | MH999592 |
| 1704256 | Arrecife do Farol, Baia da Traição, PB, 16.II.2017 | -6.686881, -34.930664 | MH999593 |
| 1704309 | Arrecife do Farol, Baia da Traição, PB, 16.II.2017 | -6.686881, -34,930664 | MH999594 |
| 1704310 | Arrecife do Farol, Baia da Traição, PB, 16.II.2017 | -6.686881, -34.930664 | MH999595 |
| 1704313 | Arrecife do Farol, Baia da Traição, PB, 16.II.2017 | -6.686881, -34.930664 | MH999596 |
| 1704363 | Arrecife do Farol, Baia da Traição, PB, 16.II.2017 | -6.686881, -34.930664 | MH999597 |
| 1704315 | Arrecife do Farol, Baia da Traição, PB, 16.II.2017 | -6.686881, -34.930664 | MH999598 |
| 1704246 | Cabo Branco, João Pessoa, PB, 15.II.2017 | -7.145014, -34.806472 | MH999599 |
| 1703982 | Pontal do Cupe, Ipojuca, PE, 12.II.2017 | -8.457536, -39.982994 | MH999601 |
| 1703984 | Pontal do Cupe, Ipojuca, PE, 12.II.2017 | -8.457536, -39.982994 | MH999602 |
| 1703985 | Pontal do Cupe, Ipojuca, PE, 12.II.2017 | -8.457536, -39.982994 | MH999603 |
| 1704012 | Pedra do Xáreu, Cabo de Santo Agostinho, PE, 12.II.2017 | -8.302775, -34.947014 | MH999604 |
| 1704023 | Ponta de Pedra, Goiana, PE, 13. II. 2017 | -7.629164, -34.808539 | MH999600 |
| 1704393A | Pontal do Cupe, Ipojuca, PE, 12.II.2017 | -8.457536, -39.982994 | MH999605 |
| 1704393B | Pontal do Cupe, Ipojuca, PE, 12.II.2017 | -8.457536, -39.982994 | MH999606 |
| 1704300A | Pontal do Cupe, Ipojuca, PE, 12.II.2017 | -8.457536, -39.982994 | MH999607 |
| 1704300B | Pontal do Cupe, Ipojuca, PE, 12.II.2017 | -8.457536, -39.982994 | MH999608 |
| 1704394 | Pontal do Cupe, Ipojuca, PE, 12.II.2017 | -8.457536, -39.982994 | MH999609 |
| 1704049 | Barra de São Miguel, Maceió, AL, 20.VIII.2016 | -9.840211, -35.889522 | MH999610 |
| 1704258 | Barra de São Miguel, Maceió, AL, 20.VIII.2016 | -9.840211, -35.889522 | MH999611 |
| 1704259 | Barra de São Miguel, Maceió, AL, 20.VIII.2016 | -9.840211, -35.889522 | MH999612 |
| 1704286 | Barra de São Miguel, Maceió, AL, 20.VIII.2016 | -9.840211, -35.889522 | MH999617 |
| 1704287 | Barra de São Miguel, Maceió, AL, 20.VIII.2016 | -9.840211, -35.889522 | MH999618 |
| 1704290 | Barra de São Miguel, Maceió, AL, 20.VIII.2016 | -9.840211, -35.889522 | MH999619 |
| 1704291 | Barra de São Miguel, Maceió, AL, 20.VIII.2016 | -9.840211, -35.889522 | MH999620 |
| 1704294 | Barra de São Miguel, Maceió, AL, 20.VIII.2016 | -9.840211, -35.889522 | MH999621 |
| 1704305 | Barra de São Miguel, Maceió, AL, 20.VIII.2016 | -9.840211, -35.889522 | MH999613 |
| 1704414 | Barra de São Miguel, Maceió, AL, 20.VIII.2016 | -9.840211, -35.889522 | MH999614 |
| 1704415 | Barra de São Miguel, Maceió, AL, 20.VIII.2016 | -9.840211, -35.889522 | MH999615 |
| 1704306 | Barra de São Miguel, Maceió, AL, 20.VIII.2016 | -9.840211, -35.889522 | MH999616 |
| 1703990 | Pituba Ponta de Pedra, Salvador, BA, 28.X.2015 | -13.007275, -38,454197 | MH999622 |
| 1703993 | Pituba Ponta de Pedra, Salvador, BA, 28.X.2015 | -13.007275, -38,454197 | MH999623 |
| 1704217A | Barra, Salvador, BA, 16.VIII.2016 | -13.010208, -38.520697 | MH999624 |
| 1704217B | Barra, Salvador, BA, 16.VIII.2016 | -13.010208, -38.520697 | MH999625 |
| 1704218 | Barra, Salvador, BA, 16.VIII.2016 | -13.010208, -38.520697 | MH999626 |
| 1704374 | Barra, Salvador, BA, 16.VIII.2016 | -13.010208, -38.520697 | MH999627 |
| 1704375 | Barra, Salvador, BA, 16.VIII.2016 | -13.010208, -38.520697 | MH999628 |
| 1704376 | Barra, Salvador, BA, 16.VIII.2016 | -13.010208, -38.520697 | MH999629 |
| 1704318 | Barra, Salvador, BA, 16.VIII.2016 | -13.010208, -38.520697 | MH999630 |
| 1600666 | Praia do Resende, Itacaré, BA, 30.X.2015 | -14.283958, -38.984781 | MH999631 |
| 1600668 | Praia do Resende, Itacaré, BA, 30.X.2015 | -14.283958, -38.984781 | MH999632 |
| 1600677 | Praia de Olivença, Olivença, BA, 31.X.2015 | -14.923994, -39.017847 | MH999633 |
| 1600678 | Praia de Olivença, Olivença, BA, 31.X.2015 | -14.923994, -39,017847 | MH999634 |
| 1600680 | Praia de Olivença, Olivença, BA, 31.X.2015 | -14.923994, -39,017847 | MH999635 |
| 1704230 | Praia do Resende, Itacaré, BA, 30.X.2015 | -14.283958, -38.984781 | MH999636 |
| 1704231 | Praia do Resende, Itacaré, BA, 30.X.2015 | -14.283958, -38.984781 | MH999637 |
| 1704198A | Praia do Moreira, Cumuruxatiba, Prado, BA, 5.I.2015 | -17.063500, -39.170389 | MH999638 |
| 1704198B | Praia do Moreira, Cumuruxatiba, Prado, BA, 5.I.2015 | -17.063500, -39.170389 | MH999639 |
| 1704234A | Praia do Moreira, Cumuruxatiba, Prado, BA, 5.I.2015 | -17.063500, -39.170389 | MH999640 |
| 1704234B | Praia do Moreira, Cumuruxatiba, Prado, BA, 5.I.2015 | -17.063500, -39.170389 | MH999641 |
| 1704235 | Praia do Moreira, Cumuruxatiba, Prado, BA, 5.I.2015 | -17.063500, -39.170389 | MH999642 |
| 1300479 | Enseada das Garças, Fundão,ES, 10.VIII.2014 | -20.031994, -40,159044 | MH999643 |
| 1704392 | Praia Grande, Fundão, ES, 06.X.2014 | -20.038350, -40.177894 | MH999644 |
| 1704327A | Praia Grande, Fundão, ES, 06.X.2014 | -20.038350, -40.177894 | MH999645 |
| 1704327B | Praia Grande, Fundão, ES, 06.X.2014 | -20.038350, -40.177894 | MH999646 |
| 1704328 | Praia Grande, Fundão, ES, 06.X.2014 | -20.038350, -40.177894 | MH999647 |
| 1704329 | Praia Grande, Fundão, ES, 06.X.2014 | -20.038350, -40.177894 | MH999648 |
| 1704355 | Praia Grande, Fundão, ES, 06.X.2014 | -20.038350, -40.177894 | MH999649 |
| 1704356 | Praia Grande, Fundão, ES, 06.X.2014 | -20.038350, -40.177894 | MH999650 |
| 1704357 | Praia Grande, Fundão, ES, 06.X.2014 | -20.038350, -40.177894 | MH9996511 |
| 1301026 | Barra de São João, Casimiro de Abreu, RJ, | -22.598858, -41.990372 | MH999652 |
| 1301114 | Praia do Forno, Arraial do Cabo, RJ, 19.III.2015 | -22.964136, -42.012556 | MH999653 |
| 1301115 | Praia do Forno, Arraial do Cabo, RJ, 19.III.2015 | -22.964136, -42.012556 | MH999654 |
| 1600635 | Praia do Forno, Arraial do Cabo, RJ, 19.III.2015 | -22.964136, -42.012556 | MH999655 |
| 1600637 | Praia do Forno, Arraial do Cabo, RJ, 19.III.2015 | -22.964136, -42.012556 | MH999656 |
| 1600638 | Praia do Forno, Arraial do Cabo, RJ, 19.III.2015 | -22.964136, -42.012556 | MH999657 |
| 1600639 | Praia do Forno, Arraial do Cabo, RJ, 19.III.2015 | -22.964136, -42.012556 | MH999658 |
| 1600640 | Praia do Forno, Arraial do Cabo, RJ, 19.III.2015 | -22.964136, -42.012556 | MH999659 |
| 1600654 | Praia dos Anjos, Arraial do Cabo, RJ, 17.III.2015 | -22.979344, -42,019681 | MH999660 |
| 1600655 | Praia dos Anjos, Arraial do Cabo, RJ, 17.III.2015 | -22.979344, -42,019681 | MH999661 |
| 1600661 | Praia do Forno, Arraial do Cabo, RJ, 19.III.2015 | -22.964136, -42.012556 | MH999662 |
| 1704227 | Prainha, Arraial do Cabo, RJ, 16.III.2015 | -22.961711, -42,020800 | MH999663 |
| 174228A | Prainha, Arraial do Cabo, RJ, 16.III.2015 | -22.961711, -42,020800 | MH999664 |
| 174228B | Prainha, Arraial do Cabo, RJ, 16.III.2015 | -22.961711, -42.020800 | MH999665 |
| 1600620 | Praia Preta, São Sebastião, SP, 09.III.2016 | -23.821972, -45.410717 | MH999666 |
| 1700734 | Praia das Pitangueiras, São Sebastião, SP, 09.III.2016 | -23.821972, -45.410717 | MH999667 |
| 1700735 | Praia das Pitangueiras, São Sebastião, SP, 09.III.2016 | -23.821972, -45.410717 | MH999668 |
| 1700736 | Praia das Pitangueiras, São Sebastião, SP, 09.III.2016 | -23.821972, -45.410717 | MH999669 |
| 1700737 | Praia das Pitangueiras, São Sebastião, SP, 09.III.2016 | -23.821972, -45.410717 | MH999670 |
| 1704321 | Praia das Pitangueiras, São Sebastião, SP, 09.III.2016 | -23.821972, -45.410717 | MH999671 |
| 1704323 | Praia das Pitangueiras, São Sebastião, SP, 09.III.2016 | -23.821972, -45.410717 | MH999672 |
| 1300507 | Praia do Quilombo, Penha, SC, 05.II.2015 | -26.774389, -48,6365833 | MH999673 |
| 1600594 | Praia das Cabeçudas, Itajaí, SC, 06.II.2015 | -26,924150, -48,634781 | MH999674 |
| 1600595 | Praia das Cabeçudas, Itajaí, SC, 06.II.2015 | -26,924150, -48,634781 | MH999675 |
| 1600596 | Praia das Cabeçudas, Itajaí, SC, 06.II.2015 | -26,924150, -48,634781 | MH999676 |
| 1600597 | Praia das Cabeçudas, Itajaí, SC, 06.II.2015 | -26,924150, -48,634781 | MH999677 |
| 1600598 | Praia das Cabeçudas, Itajaí, SC, 06.II.2015 | -26,924150, -48,634781 | MH999678 |
| 1600599 | Praia das Cabeçudas, Itajaí, SC, 06.II.2015 | -26,924150, -48,634781 | MH999679 |
| 1600601 | Praia da Armação, Armação, SC, 05.II.2017 | -26,780750, -48,603583 | MH999680 |
| 1600602 | Praia da Armação, Armação, SC, 05.II.2017 | -26,780750, -48,603583 | MH999681 |
| 1600603 | Praia da Armação, Armação, SC, 05.II.2017 | -26,780750, -48,603583 | MH999682 |
| 1600605 | Praia da Armação, Armação, SC, 05.II.2017 | -26,780750, -48,603583 | MH999683 |
| 1600606 | Praia da Armação, Florianópolis, SC, 07.II.2015 | -27,750419, -48,499997 | MH999684 |
| 1301096 | Parque da Guarita, Torres, RS, 03.II.2015 | -29.358381, -49.733639 | MH999685 |
| 1301097 | Parque da Guarita, Torres, RS, 03.II.2015 | -29.358381, -49.733639 | MH999686 |
| 1301098 | Parque da Guarita, Torres, RS, 03.II.2015 | -29.358381, -49.733639 | MH999687 |
| 1301099 | Parque da Guarita, Torres, RS, 03.II.2015 | -29.358381, -49.733639 | MH999688 |
| 1600586 | Parque da Guarita, Torres, RS, 03.II.2015 | -29.358381, -49.733639 | MH999689 |
| 1600587 | Parque da Guarita, Torres, RS, 03.II.2015 | -29.358381, -49.733639 | MH999690 |
| 1600591 | Parque da Guarita, Torres, RS, 03.II.2015 | -29.358381, -49.733639 | MH999691 |
| 1600610 | Parque da Guarita, Torres, RS, 03.II.2015 | -29.358381, -49.733639 | MH999692 |
| 1600612 | Parque da Guarita, Torres, RS, 03.II.2015 | -29.358381, -49.733639 | MH999693 |
| 1600613 | Parque da Guarita, Torres, RS, 03.II.2015 | -29.358381, -49.733639 | MH999694 |
| 1600614 | Parque da Guarita, Torres, RS, 03.II.2015 | -29.358381, -49.733639 | MH999695 |

**Table S3**. Φ-st statistic calculated using the Tamura and Nei method for *Agauopsis legionium* species complex. Non-significant Φ-st are in bold (significance level=0.05).

N01 NE2 NE3 NE5 E01 E02 E04 SE1 SE2 SE3 SE4

N01 0.00000

NE2 0.89242 0.00000

NE3 0.88192 0.59907 0.00000

NE5 0.71481 0.25331 0.26809 0.00000

E01 0.87225 0.86887 0.83775 0.51543 0.00000

E02 0.87576 0.88705 0.86226 0.48914 0.44859 0.00000

E04 0.85452 0.87685 0.85956 0.56794 0.81052 0.83282 0.00000

SE1 0.80991 0.81883 0.76620 0.45589 0.69987 0.70381 0.18103 0.00000

SE2 0.73295 0.72201 0.67598 0.42266 0.57114 0.53937 0.18611 **0.05790** 0.00000

SE3 0.78464 0.65316 0.57857 0.19198 0.48704 0.41666 0.50931 0.33934 0.26973 0.00000

SE4 0.74800 0.71309 0.64779 0.38237 0.51065 0.44117 0.40835 **0.21403 0.08646 0.07190** 0.00000

**Table S4**. Φ-st statistic calculated using the Tamura and Nei method for *Rhombognthus levigatoides* populations. Non-significant Φ-st are in bold (significance level=0.05)

NE1 NE2 NE3 NE4 NE5 NE6 E01 E02 E03 E04 SE1 SE2 SE3 RG1

NE1 0.00000

NE2 0.40460 0.00000

NE3 0.57463 **0.02715** 0.00000

NE4 **0.20577** **0.08556** 0.13075 0.00000

NE5 0.63013 0.51697 0.64493 0.25152 0.00000

NE6 0.62503 0.54297 0.65019 0.28886 0.35673 0.00000

E01 0.76401 0.75249 0.78559 0.54037 0.80718 0.80612 0.00000

E02 0.81877 0.79080 0.83034 0.56803 0.86066 0.85579 0.31417 0.00000

E03 0.94525 0.92481 0.94515 0.83910 0.95740 0.95398 0.87504 0.89870 0.00000

E04 0.95313 0.93674 0.95100 0.87278 0.96023 0.95748 0.89356 0.91716 0.88864 0.00000

SE1 0.84633 0.84956 0.85973 0.80207 0.86550 0.86953 0.82202 0.83188 0.72434 0.54556 0.00000

SE2 0.90420 0.89682 0.91370 0.82272 0.92587 0.92625 0.85826 0.87503 0.81984 0.75014 0.13997 0.00000

SE3 0.88304 0.88113 0.89339 0.82473 0.90151 0.90358 0.85086 0.86353 0.78336 0.67177 0.08675 0.14125 0.00000

RG4 0.87079 0.87140 0.88394 0.81367 0.89269 0.89602 0.83433 0.84951 0.75722 0.56704 0.09289 0.24089 0.16015 0.00000
