# Supplementary material for: Evaluating the boundaries of marine biogeographic regions of the Southwestern Atlantic using halacarid mites (Halacaridae), meiobenthic organisms with a low dispersal potential
Source: Ecol Evol. 2019 Nov 7;9(23):13359–74. doi: 10.1002/ece3.5791 (PMC6912894; doi:10.1002/ece3.5791)
Supplement: Supplementary file 4 [file ECE3-9-13359-s004.docx]

**Appendix 4: Niche comparison and modeling**

In this appendix, we present the results from some analyses accessory to those presented in the main text and provide the R scripts employed to perform them. It is largely based on R package manuals and vignettes, but we consider that presenting them here may help future researchers interested in using these packages.

Script 1. R script for checking layers for correlation.

#For installing the ENMTools package (skip if already installed)

install.packages("devtools")

library(devtools)

install_github("danlwarren/ENMTools")

#load libraries

library(ENMTools)

#Create a stack from the ascii files. Adjust the path to the folder according to your computer

env.files <- list.files(path = "C:/Users/Dell/Documents/layers", full.names = TRUE)

env <- stack(env.files)

crs(env) <- " +proj=longlat +datum=WGS84 +ellps=WGS84 +towgs84=0,0,0"

#It is optional, you may want plot the layers to check them individually

plot(env)

#Calculate the Pearson Correlation Matrix

cor_matrix<-raster.cor.matrix(env)

#Plot a heat map with the results

cor_plots<-raster.cor.plot(env)

#write a file with a table including the values

write.table(cor_matrix, file = "table_correlation.csv")

Table 1. Layers considered for Enviromental Niche Modeling analyses and highly correlated (Pearson correlation coefficient module larger than 0.75 ) variables excluded from further consideration.

| Included Layers (>0.75) | Definition and hightly correlated Layers |
| --- | --- |
| Bathymetry (MARSPEC) | Depth of the seafloor (m). |
| Bio2 (ECOCLIMATE) | Mean diurnal range (°C) (mean of monthly (max temp min temp)). |
| Bio3 (ECOCLIMATE) | Isothermality (%) (100*Bio2/Bio7). |
| Bio12 (ECOCLIMATE) | Annual precipitation (mm/m^2^). Bio13 (ECOCLIMATE): Precipitation of wettest month (mm/m^2^), Bio16 (ECOCLIMATE): Precipitation of wettest quarter (mm/m^2^). |
| Bio18 (ECOCLIMATE) | Precipitation of warmest quarter (mm/m^2^) |
| Bio19 (ECOCLIMATE) | Precipitation of coldest quarter (mm/m^2^) |
| Biogeo1 (MARSPEC) | East/West Aspect (radians) |
| Biogeo2 (MARSPEC) | North/South Aspect (radians) |
| Biogeo3 (MARSPEC) | Plan Curvature |
| Biogeo4 (MARSPEC) | Profile Curvature; Biogeo7 (MARSPEC) Concavity (degrees); Biogeo6 (MARSPEC): Bathymetric Slope (degrees); |
| Biogeo9 (MARSPEC) | Sea Surface Salinity (SSS) of the freshest month (psu). Biogeo8 (MARSPEC): Mean Annual SSS (psu); Biogeo11, Annual range in SSS (psu) Salinity maxn (Bio-Oracle) (PSS); Salinity mean (Bio-Oracle) (PSS); Chlorophyll A min. (Bio-Oracle); Chlorophyll A min(mg/m³). Phytoplankton min (mmol/m^3^); Primary productivity mean (g/m^3^day); Primary productivity min (g/m^3^day); Silicate max. (Bio-Oracle): Silicate max. concentration (mmol/m^3^). Biogeo11 (MARSPEC): Annual range in SSS (psu); Biogeo12 (MARSPEC): Annual variance in SSS (psu); Dissolved Iron max. (mmol/m^3^); Dissolved Iron mean (mmol/m^3^); Dissolved Iron min. (mmol/m^3^); Nitrate concentration mean (mmol/m^3^); Salinity mean (Bio-Oracle) (PSS); Salinity min (Bio-Oracle) (PSS); Silicate min concentration (Bio-Oracle) (mmol/m^3^); Silicate mean concentration (Bio-Oracle) (mmol/m^3^); pH (Bio-Oracle) Sea water pH. |
| Biogeo10 (MARSPEC) | SSS of the saltiest month (psu) |
| Calcite concentration (Bio-Oracle) | Mean Calcite concentration (mol/m³) |
| Chlorophyll A mean (Bio-Oracle) | Chlorophyll A mean (mg/m³). Chlorophyll A max (Bio-Oracle), Phytoplankton max (mmol/m^3^); Phytoplankton mean (mmol/m^3^); Primary productivity max (g/m^3^day). |
| Cloud cover mean (Bio-Oracle) | Cloud cover mean (%). Cloud cover max (Bio-Oracle): Cloud cover max (%). |
| Cloud cover min. | Cloud cover min (%). |
| Current velocity max. (Bio-Oracle) | Current velocity max (m/s). |
| Current velocity mean (Bio-Oracle) | Current velocity mean (m/s). Current velocity min (m/s) (Bio-Oracle) |
| Diffuse attenuation max. (Bio-Oracle) | Diffuse attenuation max. (m^-1^). Diffuse attenuation min. (m^-1^). |
| Light at the bottom max. (Bio-Oracle) | Light at the bottom max. (Einstein/m²day). |
| Light at the bottom min. (Bio-Oracle) | Light at the bottom min. (Einstein/m²day). |
| Nitrate min. (Bio-Oracle) | Nitrate concentration min. (mmol/m^3^). Silicate concentration min. (mmol/m^3^); Salinity max. (Bio-Oracle ) (PSS). Salinity mean (Bio-Oracle (PSS). |
| Photosynthetically Active radiation max (Bio-Oracle) | Photosynthetically Active radiation max (Einstein/m²/day) |
| Sea Surface Temperature mean (Bio-Oracle) | Sea Surface Temperature mean (°C). Bio1 (ECOCLIMATE): Annual mean temperature (°C); Bio10 (ECOCLIMATE): Mean temperature of warmest quarter (°C); Bio11 (ECOCLIMATE): Mean temperature of coldest quarter (°C); Bio14 (ECOCLIMATE): Precipitation of driest month (mm/m2); Bio15 (ECOCLIMATE): Precipitation seasonality - % (coefficient of variation); Bio 4 (ECOCLIMATE): Temperature seasonality (%) (standard deviation *100); Bio9 (ECOCLIMATE): Mean temperature of driest quarter (°C); Bio17 (ECOCLIMATE): Precipitation of driest quarter (mm/m^2^); Biogeo13 (MARSPEC): Mean Annual Sea Surface Temperature (SST, °C); Biogeo14 (MARSPEC): SST of the coldest month (°C); Biogeo15 (MARSPEC): SST of the warmest month (°C); biogeo16 (MARSPEC): Annual range in SST (°C); Biogeo17 (MARSPEC): Annual variance in Sea Surface Temperature (SST, °C); Dissolved molecular oxygen max. (mmol/m3), Photosynthetically Active radiation mean (Einstein/m²day); Sea Surface Temperature max (Bio-Oracle) (°C); Sea Surface Temperature min (Bio-Oracle) (°C). Bio6 (ECOCLIMATE): Min temperature of coldest month (°C); Bio5 (ECOCLIMATE): Max temperature of warmest month (°C); Bio7 (ECOCLIMATE): Temperature annual range (°C) (Bio5-Bio6) Bio8 (ECOCLIMATE): Mean temperature of wettest quarter (°C); Phosphate mean (Bio-Oracle): Phosphate mean concentration (μmol/l). Phosphate max. concentration (μmol/l); Phosphate min. concentration (μmol/l); Dissolved molecular oxygen min. (Bio-Oracle): Dissolved molecular oxygen min. (mmol/m3). |

Script 2. R script for pre-modelling niche comparison

#load libraries

library (ecospat)

library (ENMTools)

library (raster)

#Occurrence data

# Load the occurrence records. In our case, both sibling species of *Rhombognathus*

LonLatDataNE <- read.csv("Rhombognathus_NE.csv")[,2:3]

LonLatDataSE <- read.csv("Rhombognathus_SE.csv")[,2:3]

#Then load the environmental variables into R with the help of the stack function of the 'raster' package. #You can not just copy the following line but have to adjust the filepath to your own.

files <- list.files("C:/Users/Dell/Documents/layers.75",pattern='asc',full.names=TRUE)

Grids <- raster::stack(files)

#Create a background for each species to emulate a known distribution

env.files <- list.files(path = "C:/Users/Dell/Documents/layers.75", full.names = TRUE)

env <- stack(env.files)

env <- setMinMax(env)

crs(env) <- " +proj=longlat +datum=WGS84 +ellps=WGS84 +towgs84=0,0,0"

NE_SP <- enmtools.species()

NE_SP$species.name <- "NE"

NE_SP$presence.points <- LonLatDataNE

NE_SP$range <- background.raster.buffer(NE_SP$presence.points, 20000, mask = env)

NE_SP$background.points <- background.points.buffer(points = NE_SP$presence.points, radius = 20000, n = 500, mask = env[[1]])

#take a look on the results

plot (NE_SP)

SE_SP <- enmtools.species()

SE_SP$species.name <- "SE"

SE_SP$presence.points <- LonLatDataSE

SE_SP$range <- background.raster.buffer(SE_SP$presence.points, 20000, mask = env)

SE_SP$background.points <- background.points.buffer(points = SE_SP$presence.points, radius = 20000, n = 500, mask = env[[1]])

#take a look on the results

plot (SE_SP)

#Extract variables from each species range

VariablesAtNE_SPbg <- raster::extract(Grids,NE_SP$background.points)

VariablesAtSE_SPbg <- raster::extract(Grids,SE_SP$background.points)

#Values for background points for each species

OutputNE_SPbg <- as.data.frame(cbind("species", NE_SP$background.points, VariablesAtNE_SPbg))

colnames(OutputNE_SPbg) <- c("species","x","y", colnames(VariablesAtNE_SPbg))

OutputSE_SPbg <- as.data.frame(cbind("species", SE_SP$background.points, VariablesAtSE_SPbg))

colnames(OutputSE_SPbg) <- c("species","x","y", colnames(VariablesAtSE_SPbg))

# Extracting the variables for all occurrencelocations

VariablesAtOccurrencelocationsNE <- raster::extract(Grids,LonLatDataNE)

VariablesAtOccurrencelocationsSE <- raster::extract(Grids,LonLatDataSE)

# Combining the extracted values with the longitude and latitude values

NE_POINTS <- as.data.frame(cbind("Rhombognathus_NE", LonLatDataNE, VariablesAtOccurrencelocationsNE))

SE_POINTS <- as.data.frame(cbind("Rhombognathus_SE", LonLatDataSE, VariablesAtOccurrencelocationsSE))

colnames(NE_POINTS) <- c("species","x","y", colnames(VariablesAtOccurrencelocationsNE))

colnames(SE_POINTS) <- c("species","x","y", colnames(VariablesAtOccurrencelocationsSE))

#Complete dataset

NE<-rbind(NE_POINTS,OutputNE_SPbg)

NE <-NE[complete.cases(NE), ]

SE<-rbind(SE_POINTS,OutputSE_SPbg)

SE <-SE[complete.cases(SE), ]

#PCA.env score NE, SE and niche comparison

pca.env <- dudi.pca(rbind(NE,SE)[,4:ncol(NE)],scannf=F,nf=2)

#plot a correlation circle with PCA results

ecospat.plot.contrib(contrib=pca.env$co, eigen=pca.env$eig)

#Save variables correlations to principal components

write.table(pca.env$co, file = "corr_pca.csv")

# PCA scores for the whole study area

scores.globclim <- pca.env$li

# PCA scores for the species NE distribution

scores.sp.NE <- suprow(pca.env,NE[which(NE[,1]=="Rhombognathus_NE"),4:ncol(NE)])$li

# PCA scores for the species SE distribution

scores.sp.SE <- suprow(pca.env,SE[which(SE[,1]=="Rhombognathus_SE"),4:ncol(SE)])$li

# PCA scores for the whole NE study area

scores.clim.NE <- suprow(pca.env,NE[,4:ncol(NE)])$li

# PCA scores for the whole SE study area

scores.clim.SE <- suprow(pca.env,SE[,4:ncol(SE)])$li

# gridding the NE niche

grid.clim.NE <- ecospat.grid.clim.dyn(glob=scores.globclim,

glob1=scores.clim.NE,

sp=scores.sp.NE, R=100,

th.sp=0)

# gridding the SE niche

grid.clim.SE <- ecospat.grid.clim.dyn(glob=scores.globclim,

glob1=scores.clim.SE,

sp=scores.sp.SE, R=100,

th.sp=0)

# Compute Schoener's D, index of niche overlap

D.overlap <- ecospat.niche.overlap (grid.clim.NE, grid.clim.SE, cor=T)$D

D.overlap

#Delimiting niche categories and quantifying niche dynamics in analogue climates

#with ecospat.niche.dyn.index()

niche.dyn <- ecospat.niche.dyn.index (grid.clim.NE, grid.clim.SE, intersection = 0.1)

ecospat.plot.niche.dyn(grid.clim.NE, grid.clim.SE, quant=0.25, interest=2,

title= "Niche Overlap", name.axis1="PC1",

name.axis2="PC2")

ecospat.shift.centroids(scores.sp.NE, scores.sp.SE, scores.clim.NE, scores.clim.SE)

#Perform the Niche Equivalency Test according to Warren et al. (2008)

#Niche equivalency test H1: Is the overlap between the NE and SE niches higher than two random niches?

eq.testNESE <- ecospat.niche.equivalency.test(grid.clim.NE, grid.clim.SE,

rep=100, alternative = "greater")

ecospat.plot.overlap.test(eq.testNESE, "D", "Equivalency")

Table 2. Correlation of each variable relative to the two first Principal Components of A. legionium X *R. levigatoides* Southeastern Clade (Component 1 = 21.80 %, Component 2 = 18.65 % of variability), A. legionium X *R. levigatoides* Northeastern Clade (Component 1 = 19.83 %, Component 2 = 19.12 % of variability), and Southeastern and Northeastern of *R. levigatoides* complex (Component 1 = 23.92 %, Component 2 = 17.53 % of variability).

|  | *A. legionium X*  *R. levigatoides* SE | | *A. legionium X*  *R. levigatoides* NE | | *R. levigatoides*  NEXSE | |
| --- | --- | --- | --- | --- | --- | --- |
|  | Comp1 | Comp2 | Comp1 | Comp2 | Comp1 | Comp2 |
| Bathymetry | -0,55 | -0,13 | -0,51 | -0,43 | 0,02 | -0,71 |
| Mean diurnal range | -0,21 | -0,24 | -0,42 | -0,25 | 0,03 | -0,40 |
| Isothermality | -0,46 | -0,76 | -0,88 | -0,03 | -0,57 | -0,54 |
| Annual precipitation | -0,85 | 0,12 | -0,54 | -0,59 | 0,32 | -0,69 |
| Precipitation of warmest quarter | -0,49 | 0,31 | -0,22 | -0,06 | -0,01 | -0,47 |
| Precipitation of coldest quarter | -0,76 | 0,19 | -0,22 | -0,19 | 0,04 | -0,24 |
| East/West Aspect | 0,05 | 0,20 | 0,11 | 0,30 | -0,13 | -0,10 |
| North/South Aspect | -0,63 | -0,29 | -0,66 | -0,17 | -0,20 | -0,52 |
| Plan Curvature | -0,09 | 0,13 | -0,19 | -0,27 | 0,12 | -0,32 |
| Profile Curvature | -0,08 | 0,09 | -0,28 | -0,38 | 0,11 | -0,40 |
| Sea Surface Salinity of freshest month | 0,71 | -0,42 | 0,04 | 0,69 | -0,85 | 0,25 |
| SSS of the saltiest month | 0,09 | -0,73 | -0,34 | 0,45 | -0,79 | 0,39 |
| Mean Calcite concentration | -0,59 | -0,36 | -0,49 | -0,48 | 0,04 | -0,54 |
| Chlorophyll A mean | -0,02 | 0,65 | 0,45 | -0,69 | 0,86 | 0,03 |
| Cloud cover mean | -0,65 | -0,07 | -0,22 | 0,11 | -0,35 | -0,31 |
| Cloud cover min. | -0,43 | 0,06 | 0,00 | 0,36 | -0,33 | -0,12 |
| Current velocity max. | -0,23 | -0,38 | -0,48 | 0,62 | -0,59 | -0,40 |
| Current velocity mean | 0,14 | -0,70 | -0,55 | 0,31 | -0,62 | -0,15 |
| Diffuse attenuation max. | -0,80 | 0,08 | -0,33 | -0,72 | 0,48 | -0,56 |
| Light at the bottom max. | -0,35 | -0,07 | -0,33 | -0,24 | 0,03 | -0,54 |
| Light at the bottom min. | -0,31 | -0,54 | -0,71 | 0,04 | -0,46 | -0,67 |
| Nitrate min. | -0,40 | 0,56 | 0,28 | -0,47 | 0,60 | -0,20 |
| Photosynthetically Active radiation max | 0,36 | -0,48 | -0,19 | 0,77 | -0,73 | 0,15 |
| Sea Surface Temperature mean | -0,19 | -0,89 | -0,78 | 0,46 | -0,91 | -0,16 |

Script 3. R script for setting the feature numbers and regularization

In this case, note that we performed two classes of analyses, with and without shaping the background according to the sampling bias.

#load libraries

library (ENMeval)

library(ENMTools)

#read enviromental layers

env.files.NE <- list.files(path = "C:/Users/Dell/Documents/layers.NE", full.names = TRUE)

env.NE <- stack(env.files.NE)

env.NE <- setMinMax(env.NE)

crs(env.NE) <- " +proj=longlat +datum=WGS84 +ellps=WGS84 +towgs84=0,0,0"

#If you want take a look on them

plot(env.NE)

#read occurrences

Rhus_NE_occ <- read.csv("Rhombognathus_NE.csv")[,2:3]

#evaluate NE, no sampling bias (You may look at manual to change according your #application)

enmeval_Rhus_NE <- ENMevaluate(Rhus_NE_occ, env.NE, method="jackknife", n.bg=500, RMvalues = seq(0.5, 3, 0.5), fc = c("L", "LQ"), algorithm='maxent.jar')

#Saving results

par(mfrow=c(2,2))

eval.plot(enmeval_Rhus_NE@results, "AICc")

dev.copy(pdf,'AICc_enmeval_Rhus_NE.pdf')

dev.off()

eval.plot(enmeval_Rhus_NE@results, "avg.test.AUC", variance="var.test.AUC")

dev.copy(pdf,'avgtestAUC_enmeval_Rhus_NE.pdf')

dev.off()

aic.mod <- enmeval_Rhus_NE@models[[which(enmeval_Rhus_NE@results$delta.AICc==0)]]

enmeval_Rhus_NE_varimpbest<-var.importance(aic.mod)

write.table(enmeval_Rhus_NE_varimpbest, file = "enmeval_Rhus_NE_varimpbest.csv")

enmeval_Rhus_NE_varimp<-lapply(enmeval_Rhus_NE@models, var.importance)

write.table(enmeval_Rhus_NE_varimp, file = "enmeval_Rhus_NE_varimp.csv")

plot(enmeval_Rhus_NE@predictions[[which (enmeval_Rhus_NE@results$delta.AICc == 0) ]])

dev.copy(pdf,'bestmodel_Rhus_NE.pdf')

dev.off()

#Now, evaluate considering bias sampling

env.files.NEwb <- list.files(path = "C:/Users/Dell/Documents/layers.NEwb", full.names = TRUE)

env.NEwb <- stack(env.files.NEwb)

env.NEwb <- setMinMax(env.NEwb)

crs(env.NEwb) <- " +proj=longlat +datum=WGS84 +ellps=WGS84 +towgs84=0,0,0"

plot(env.NEwb)

#background buffer for sampling bias

points <- read.csv("Sampling_locals.csv")[,1:2]

Sampling <- enmtools.species()

Sampling$species.name <- "Sampling"

Sampling$presence.points <- read.csv("Sampling_locals.csv")[,1:2]

Sampling$range <- background.raster.buffer(Sampling$presence.points, 25000, mask = env.NEwb)

Sampling$background.points <- background.points.buffer(points = Sampling$presence.points, radius = 25000, n = 500, mask = env.NEwb[[1]])

plot (Sampling)

plot (Sampling$background.points)

bg<-rbind (Sampling$background.points,points)

enmeval_Rhus_NEwb <- ENMevaluate(Rhus_NE_occ, env.NEwb, bg.coords = bg, method="jackknife", RMvalues = seq(0.5, 3, 0.5), fc = c("L", "LQ"), algorithm='maxent.jar')

#Saving results

par(mfrow=c(2,2))

eval.plot(enmeval_Rhus_NEwb@results, "avg.test.AUC", variance="var.test.AUC")

dev.copy(pdf,'avgtestAUC_enmeval_Rhus_NEwb.pdf')

dev.off()

eval.plot(enmeval_Rhus_NEwb@results, "AICc")

dev.copy(pdf,'AICc_enmeval_Rhus_NEwb.pdf')

dev.off()

aic.mod <- enmeval_Rhus_NEwb@models[[which(enmeval_Rhus_NEwb@results$delta.AICc==0)]]

enmeval_Rhus_NEwb_varimpbest<-var.importance(aic.mod)

write.table(enmeval_Rhus_NEwb_varimpbest, file = "enmeval_Rhus_NEwb_varimpbest.csv")

enmeval_Rhus_NEwb_varimp<-lapply(enmeval_Rhus_NEwb@models, var.importance)

write.table(enmeval_Rhus_NEwb_varimp, file = "enmeval_Rhus_NEwb_varimp.csv")

plot(enmeval_Rhus_NEwb@predictions[[which (enmeval_Rhus_NEwb@results$delta.AICc == 0) ]])

dev.copy(pdf,'bestmodel_Rhus_NEwb.pdf')

dev.off()


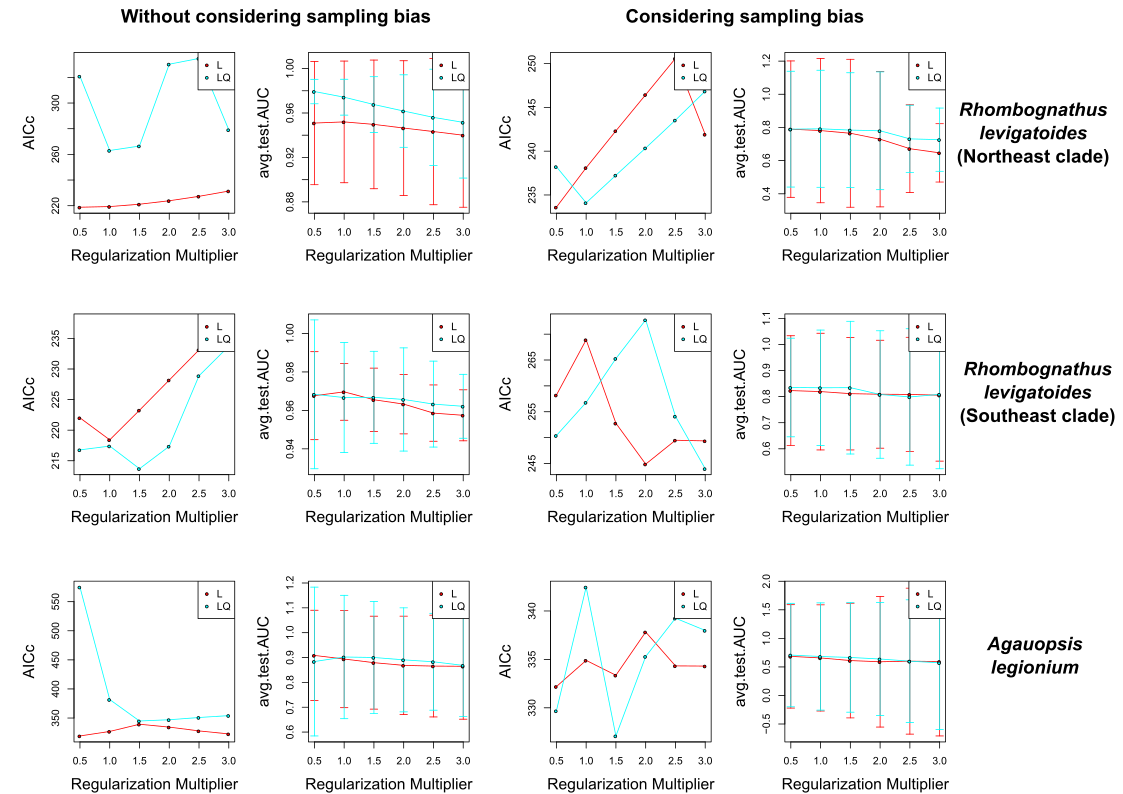


S Figure 1. ENMEval results. Plots refer to AICc values and the average of AUC Test under different values of the Regularization multiplier and Linear (red lines) and Linear and Quadratic (light blue) features.

Script 4. R script for producing a sampling bias grid for Maxent (largely taken from https://scottrinnan.wordpress.com/2015/08/31/how-to-construct-a-bias-file-with-r-for-use-in-maxent-modeling/)

#load libraries

library(raster)

library(MASS)

library(magrittr)

library(maptools)

#load sampling localities. Obviously change the path and names accordingly

locals <- read.csv("Sampling_locals.csv")

#load a layer to be used as mask. It must be cropped as your other enviromental layers

mask <- brick("bathy.tif")

#set projection etc.

crs(mask) <- " +proj=longlat +datum=WGS84 +ellps=WGS84 +towgs84=0,0,0"

#turn into a raster the occurrence points

occur.ras <- rasterize(locals, mask,1)

#Extract coordinates from the raster

presences <- which(values(occur.ras) == 1)

pres.locs <- coordinates(occur.ras)[presences, ]

#kde2d function gives us a two-dimensional kernel density estimate, based on the coordinates of the #occurrence points. Note that here we set the value of bandwidth. Here we apply a very narrow area #around sampling points. Adjust it according to your problem.

dens <- kde2d(pres.locs[,1], pres.locs[,2], 0.4, n = c(ncol(occur.ras), nrow(occur.ras)), lims = c(-52, -26, -33, 2))

dens.ras <- raster(dens)

#Plot the raster to check

plot(dens.ras)

#Adjust values and crop using the mask. Note that a valid bias grid must have no zero or negative values.

bb <- extent(-52, -26, -33, 2)

dens.ras<- setExtent(dens.ras, bb)

masked_dens.ras<-mask(dens.ras, mask)

values(masked_dens.ras)[values(masked_dens.ras) <= 1.0e-45] = 1.0e-45

#Take a look to see if everything is ok

masked_dens.ras

plot (masked_dens.ras)

#save in a grid file. Maybe you will need to convert in an ascii file.

writeRaster(masked_dens.ras, filename="C:/Maxent/bias file.grd")


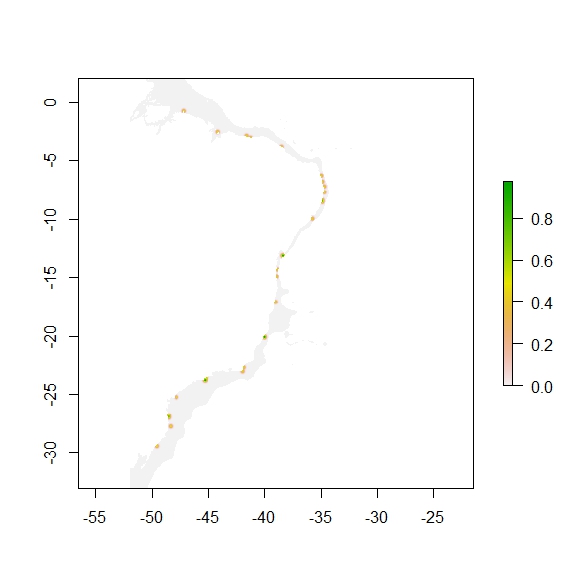


S Figure 2. Grid employed in the analyses that takes into account sampling bias.
